# Activation of autoreactive lymphocytes in the lung by radioresistant cells expressing a STING gain-of-function mutation

**DOI:** 10.1172/jci.insight.174331

**Published:** 2024-07-18

**Authors:** Kevin MingJie Gao, Kristy Chiang, Sharon Subramanian, Xihui Yin, Paul J. Utz, Kerstin Nündel, Kate A. Fitzgerald, Ann Marshak-Rothstein

**Affiliations:** 1 Division of Innate Immunity and; 2 Division of Rheumatology, Department of Medicine, UMass Chan Medical School, Worcester, Massachusetts, USA.; 3 Department of Medicine, Division of Immunology and Rheumatology, Stanford University School of Medicine, Stanford, California, USA.

**Keywords:** Autoimmunity, Inflammation, Adaptive immunity, Autoimmune diseases, Innate immunity

## Abstract

Gain-of-function mutations in the dsDNA sensing adaptor STING lead to a severe autoinflammatory syndrome known as STING-associated vasculopathy with onset in infancy (SAVI). Patients with SAVI develop interstitial lung disease (ILD) and produce autoantibodies that are commonly associated with systemic autoimmune diseases. Mice expressing the most common SAVI mutation, STING V154M (VM), similarly develop ILD but exhibit severe T and B cell lymphopenia and low serum Ig titers, and they lack autoantibodies. Importantly, lethally irradiated VM hosts reconstituted with WT stem cells (WT→VM) still develop ILD. In this study, we find that WT→VM chimeras had restored B cell function, produced autoantibodies, and thereby recapitulated the loss of tolerance seen in patients with SAVI. Lymphocytes derived from both WT and BCR or TCR transgenic (Tg) donors accumulated in the extravascular lung tissue of WT+Tg→VM mixed chimeras, but lymphocyte activation and germinal center formation required WT cells with a diverse repertoire. Furthermore, when T cells isolated from the WT→VM chimeras were adoptively transferred to naive Rag1-deficient secondary hosts, they trafficked to the lung and recruited neutrophils. Overall, these findings indicated that VM expression by radioresistant cells promoted the activation of autoreactive B cells and T cells that then differentiated into potentially pathogenic effector subsets.

## Abstract 2

Immunocompetent lymphocytes that develop within a host expressing a constitutively active STING mutation have the capacity to become autoreactive against lung tissue self-antigens.

## Graphical abstract

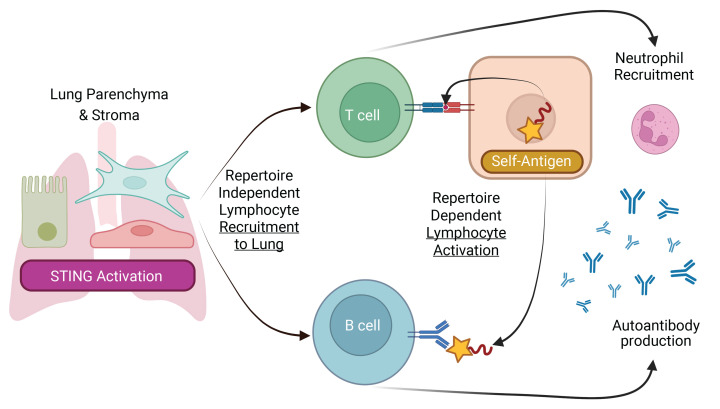

## Introduction

Autoinflammatory and autoimmune diseases exist on a continuum, reflecting the connection between innate and adaptive self-reactivity. Both can involve dysregulation of immune responses that leads to a breach in tolerance and the ensuing activation of autoreactive lymphocytes. While some diseases may originate entirely from the dysregulation of adaptive immune responses (1), many diseases commonly recognized as autoimmune can be triggered by aberrant activation of innate immune processes. For example, in the autoimmune disease systemic lupus erythematosus (SLE), inadequate clearance of cell debris in combination with activation of endosomal TLRs leads to the activation of B cells reactive to nucleic acids (NA) or NA-binding proteins (2). Similar autoantibody reactivities develop in patients with a range of autoinflammatory diseases that result from defects in NA sensing, degradation, or metabolism (3–5). One such autoinflammatory disease is STING-associated vasculopathy with onset in infancy (SAVI), a syndrome caused by constitutive ligand-independent activation of STING, the adaptor protein in the cGAS-STING cytosolic dsDNA sensing pathway. Patients with SAVI commonly develop interstitial lung disease (ILD), and in a cohort of 21 patients with SAVI, 20 were found to be positive for autoantibodies (5).

Mouse models of SAVI that express the 2 most common SAVI-causing STING mutations, STING N153S (NS) and STING V154M (VM), recapitulate many of the clinical manifestations found in patient populations, including ILD (6, 7). VM mice develop more severe ILD than NS mice and, despite the accumulation of activated extravascular (EV) B cells in the lung, they develop peripheral B cell lymphopenia much earlier in life (6, 8) and exhibit a more severe defect in B cell development and antibody production. Expression of the VM mutation in T cells is known to compromise T cell survival by directly triggering an unfolded protein response (UPR) that causes apoptotic cell death (9, 10). Analogously, STING activation in B cells has also been found to impair B cell survival (8, 11). In contrast to mouse B cells, human B cells do not express STING (12), and patients with SAVI do not show B cell lymphopenia.

We previously reported that lethally irradiated VM mice reconstituted with WT stem cells (WT→VM radiation chimeras) do not have lymphopenia and develop more extensive lung inflammation than the parental VM mice (13). These data indicate that nonhematopoietic cells expressing the VM mutation can recruit and activate WT lymphocytes and thereby play a substantial role in triggering ILD. Since lymphocyte-intrinsic VM expression is the primary driver of T and B cell lymphopenia (6), we decided to use WT→VM chimeras (13) to explore the effect of the VM host environment on potentially autoreactive WT lymphocytes. We reasoned that these chimeras would better model the autoimmune phenotype of patients with SAVI. We further incorporated BCR and TCR transgenic (Tg) mice into our model to assess the importance of self-reactivity in lymphocyte recruitment and activation in the lung. We now show that WT autoreactive B cells in a VM hosts become activated and produce autoantibodies reactive with common autoantigens as well as with lung tissue. We further found that the VM host promoted the activation of potentially self-reactive T cells and the development of long-lived lung-homing T cells. Altogether, our findings demonstrate that autoinflammation resulting from VM expression in nonhematopoietic cells triggers both B cell and T cell autoimmunity.

## Results

### B cell intrinsic VM expression impairs proliferation and antibody production.

**Figure 1:**
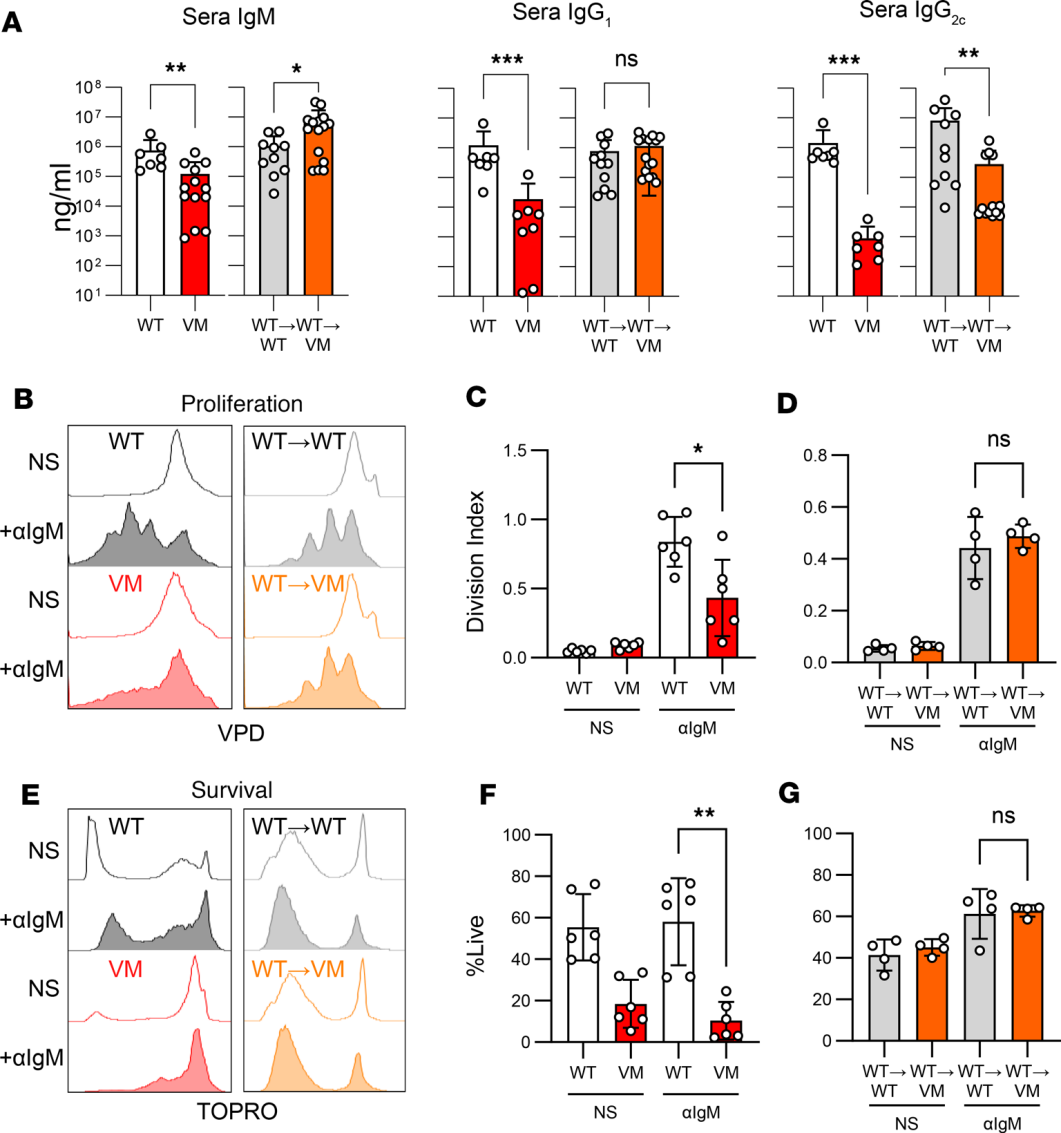
B cell intrinsic VM expression impairs antibody production, proliferation, and survival. (**A**) Serum titers of IgM, IgG_1_ and IgG_2c_ in age- and sex-matched WT (*n* = 7) and VM (*n* = 7–14) mice at 3–4 months of age, or from WT→WT (*n* = 10–11) and WT→VM (*n* = 13–15) chimeric mice at 8 weeks after reconstitution. (**B**–**G**) Splenic B220^+^ B cells from biologic replicates of WT (*n* = 6) versus VM (*n* = 6) B cells and WT→WT (*n* = 4) and WT→VM (*n* = 4) B cells labeled with violet proliferative dye (VPD) were stimulated for 72 hours with BLyS alone (NS) or anti≠IgM F(ab’)_2_ antibody and BLyS (+αIgM), and they were then stained with TOPRO to assess cell death. Cell division is shown by representative VPD histograms (**B**) and division index (**C** and **D**). Cell death is shown by representative TOPRO histograms (**E**) and percentage of TOPRO^–^ live cells (**F** and **G**). Nonparametric Mann-Whitney *U* tests were used for pair-wise comparisons to determine statistical significance (*P < 0.05, **P < 0.01, ***P < 0.001).

VM SAVI mice show lymphopenia and have been reported to have remarkably low serum antibody titers (8). Consistent with these observations, we found that VM serum IgM, IgG_1_, and IgG_2c_ titers were reduced by approximately 10-fold, 100-fold, and 1,000-fold, respectively, relative to littermate controls (Figure 1A). Low antibody titers could reflect B cell intrinsic defects in survival, proliferation, and/or differentiation, or they could reflect the effect of B cell extrinsic factors. We found that VM BM cells poorly engrafted lethally irradiated WT hosts (14), so it was not possible to directly assess the effects of BM intrinsic VM expression on B cell phenotypes. Instead, we asked whether hypogammaglobulinemia was due to the VM environment by lethally irradiating VM or WT mice and reconstituting them with WT stem cells (WT→VM and WT→WT radiation chimeras). Eight to 10 weeks later, serum Ig titers collected from WT→VM chimeras were compared with serum titers from WT→WT chimera controls. We observed a significant increase in serum IgM in WT→VM animals, a moderate reduction in IgG_2c_, and comparable titers of IgG_1_ (Figure 1A). Overall, these data indicate that low Ab titers of VM mice are not due to the VM environment and are rather due to a B cell–intrinsic defect in differentiation following BCR engagement in the setting of mutant VM STING expression (9, 11).

To test this possibility, we compared the in vitro survival and proliferative capacity of parental WT and VM B cells to B cells isolated from chimeric mice. Splenic B cells were stained with the fluorophore VPD and were then stimulated with anti-IgM F(ab’)_2_. Three days after stimulation, proliferation was assessed by VPD dilution, and viability was evaluated with TOP-RO-3 (dead cell) stain. As expected, WT B cells proliferated well in response to anti-IgM, whereas the VM B cells proliferated poorly (Figure 1, B and C) and had reduced survival, even in the presence of the B cell survival factor BLyS (Figure 1, E and F). Thus, VM B cells show an activation-induced demise precluding robust proliferation, consistent with previous studies of B cell responses to STING agonists (11) and analogous to the increased cell death observed in SAVI T cells following TCR engagement (9). By contrast, we found that B cells from the WT→VM chimeras responded comparably to control WT→WT B cells (Figure 1, B, D, E, and G). Since B cells isolated from WT→VM chimeras are unimpaired as far as their capacity to proliferate and differentiate, despite ongoing systemic lung inflammation (13), we surmised that WT→VM chimeras provide a reasonable model for exploring the recruitment of potentially autoreactive B cells to the lung in the context of SAVI ILD.

### WT→VM chimeric mice develop germinal centers in the lung.

**Figure 2:**
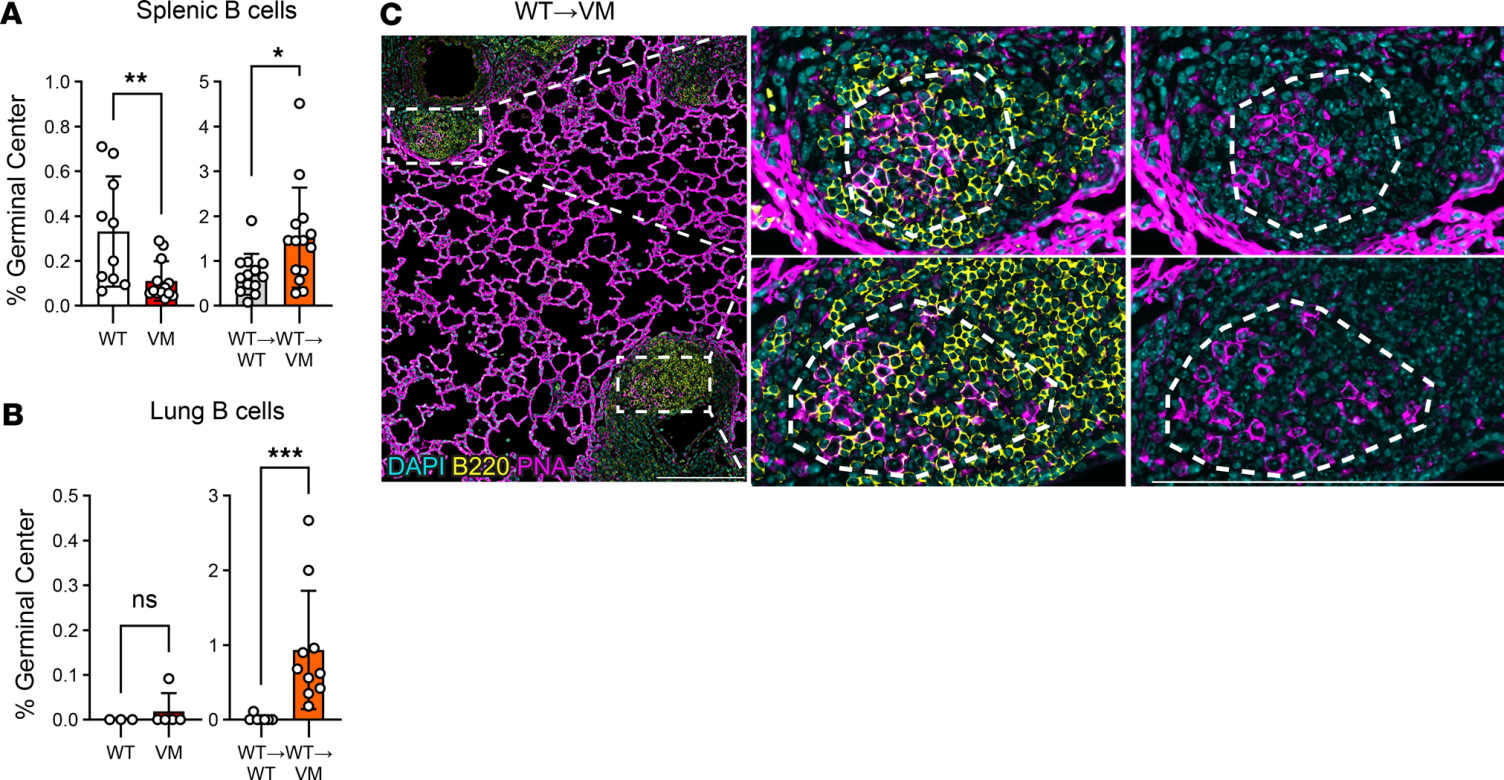
WT→VM mice form germinal centers in the lung. Data obtained from 3- to 4-month-old age- and sex-matched WT (*n* = 3–10) and VM (*n* = 5–13) mice. Chimeric WT→WT (*n* = 7–13) and WT→VM (*n* = 10–14) mice were evaluated 8–9 weeks after reconstitution. (**A**) Percentage of IgD^–^Fas^+^GL7^+^ germinal center (GC) B cells within the CD45^+^CD19^+^ B cell compartment of WT and VM spleen or within the donor-derived B cells in WT→WT and WT→VM chimeric spleens. (**B**) Percentage of GC B cells within the CD45^+^CD19^+^ lung extravascular (EV) B cell compartment of in WT and VM mice and within the total donor-derived lung EV B cells in WT→WT and WT→VM chimeras. (**C**) Immunofluorescence imaging of WT→VM chimeric mouse lungs stained for B220 (yellow) and peanut agglutinin (PNA, magenta) to identify B220^+^PNA^+^ GC B cells. Two B cell follicles containing GCs are highlighted with white squares. Magnification (4×) of the highlighted sections are shown in the 2 panels to the right, and a dotted circular outline is used to identify the germinal center. In the middle panel, DAPI, B220, and PNA are shown together, and the right panel shows DAPI and PNA alone. Data in **C** are representative of 2 biologic replicates. Nonparametric Mann-Whitney *U* tests were used for pairwise comparisons to determine statistical significance (*P < 0.05, **P < 0.01, ***P < 0.001). Scale bars: 200 μm.

Germinal centers (GCs) are a common site of B cell expansion, differentiation, and class-switch recombination (15). Unimmunized B6 WT mice spontaneously develop small numbers of GCs in the spleen and have low but detectable numbers of Fas^+^GL7^+^ GC splenic B cells (16). VM mice have fewer B cells than WT mice (6), and the percentage of GC B cells within the total B cell compartment was also reduced (Figure 2A). In contrast, the percentage of GC B cells in spleens of WT→VM chimeras was greater than either the VM mice or the WT→WT controls, reflecting the presence of activated B cells.

Well-organized bronchus associated lymphoid tissue (BALT) can be found in the inflamed lungs of patients with autoimmune diseases such as rheumatoid arthritis and Sjögren’s syndrome and can include follicles with defined GCs (17). Although both VM and WT→VM chimeric mice exhibit extensive BALT (13), only BALT from WT→VM chimeric mice contained a significant number of GC B cells (Figure 2B). GCs within the lungs of WT→VM chimeric mice were visualized by immunofluorescence staining for peanut agglutinin (PNA), a lectin that binds to GC B cells. Although PNA also binds lectin receptors on alveolar epithelia, PNA^+^ GC cells could be identified within CD45^+^B220^+^ B cell follicles (Figure 2C). Thus, immunocompetent (WT) B cells form GC in the lungs of chimeric mice when radioresistant cells express the VM mutation.

### Activation of EV B cells requires a diverse repertoire.

BALT and GCs are normally associated with antigen-driven expansion of pathogen or autoantigen-specific B cells. Based on the frequency of GCs in the WT→VM chimeric SAVI mice, we hypothesized that a lung environment expressing the VM mutation recruits and then activates autoreactive lymphocytes. Alternatively, lymphocytes could be nonspecifically recruited and activated in a repertoire-independent fashion by the inflammatory milieu present within the SAVI lung. We reasoned that mixed radiation chimeras would facilitate our ability to track the relative accumulation of repertoire-restricted (BCR Tg) and polyclonal (non-Tg) B cells into the lungs of VM host mice.

**Figure 3:**
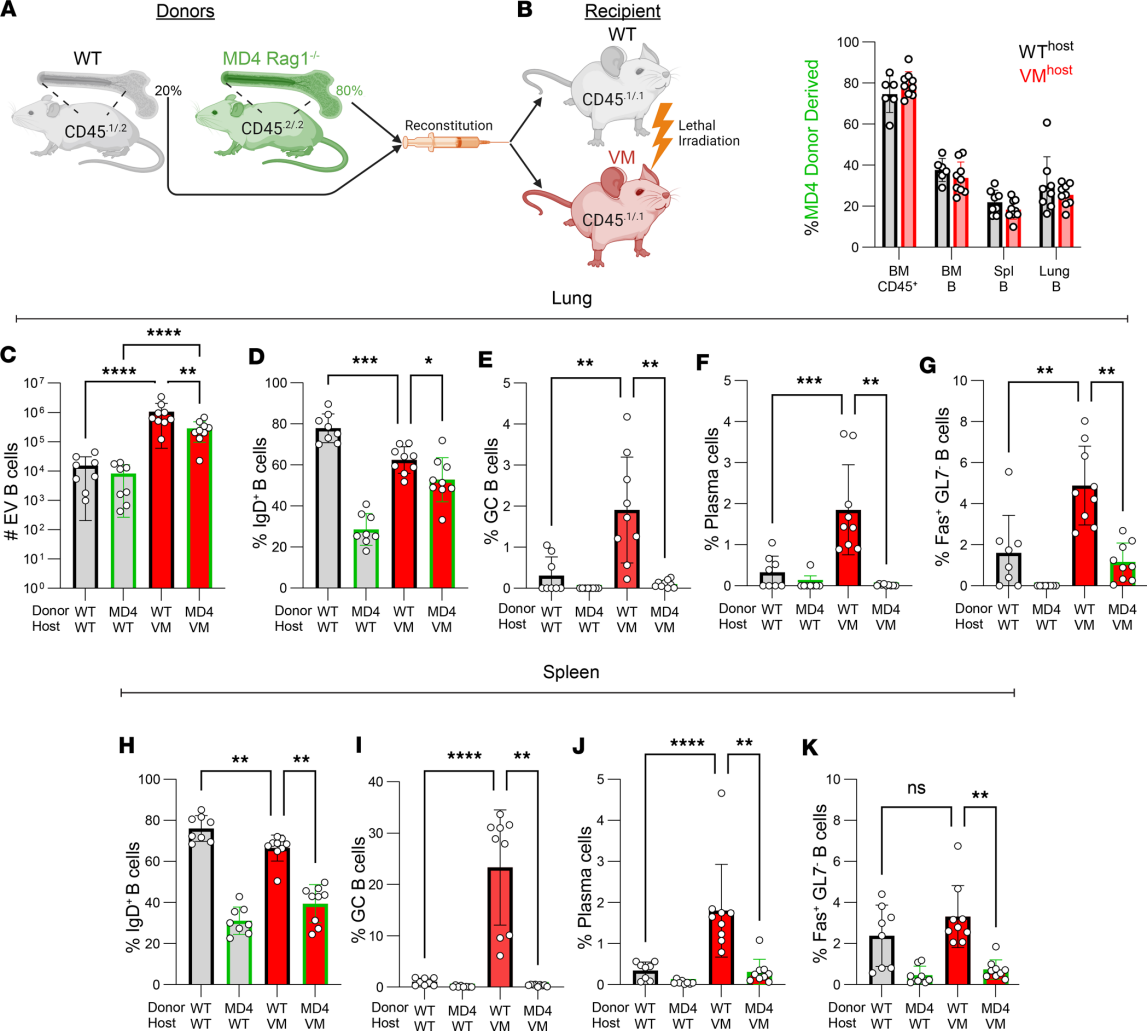
Formation of GC B cells in VM lung requires an unrestricted repertoire. (**A**) Six-week-old age- and sex-matched CD45.1/CD45.1 WT and VM littermates were lethally irradiated and reconstituted with a mixture of BM stem cells comprised of 20% CD45.1/CD45.2 WT and 80% CD45.2/CD45.2 MD4 Rag1^–/–^ cells. WT+MD4→WT (*n* = 4–8) and WT+MD4→VM (*n* = 6–9) chimeric mice were then evaluated 8–9 weeks later. (**B**) Percentage of MD4 donor–derived cells within all CD45^+^ cells in BM, BM B220^+^ B cells, or splenic CD19^+^ B cells, or within the lung extravascular (EV) B cells of either the WT hosts (gray bar) or VM hosts (red bar), after excluding any CD45.1 single-positive radio-resistant host cells. (**C**) Total number of lung EV B cells derived from either the WT or MD4 donor in either the WT or VM hosts. (**D**–**G**) Percentage of IgD^+^ naive B cell, IgD^–^ Fas^+^ GL7^+^ germinal center (GC) B cells, B220^–^ CD138^+^ PC cells, and IgD^–^ Fas^+^ GL7^–^ B cells derived from either the WT or MD4 donor within the EV B cell compartment of either WT or VM host. (**H**–**K**) Percentage of naive B cells, GC B cells, PCs, and IgD^–^Fas^+^GL7^–^ B cells derived from WT or MD4 donor within the splenic B compartment of WT or VM hosts. For **C**–**K**, data points in columns 1 and 2 represent paired WT and MD4 donors from a shared WT host. Similarly, data points in columns 3 and 4 represent paired WT and MD4 donors from a shared VM host. To determine statistical significance, nonparametric Mann-Whitney *U* tests were used for pair-wise comparisons between columns 1 and 3 and between columns 2 and 4. A Wilcoxon matched pairs signed rank test was used for pairwise comparisons between columns 3 and 4 (*P < 0.05, **P < 0.01, ***P < 0.001, ****P < 0.0001).

A mixture of CD45 allelically distinct BM stem cells from Rag1^–/–^ BCR Tg MD4 (hen egg lysozyme-specific) mice and non-Tg B6 mice at a 4:1 ratio was used to reconstitute either lethally irradiated WT mice (MD4+WT→WT chimeras) or VM mice (MD4+WT→VM chimeras), and mice were evaluated 8 weeks later (Figure 3A). The capacity of the WT- and MD4-derived stem cells to engraft the overall hematopoietic compartment was determined by examining the relative proportion of the donor populations in the BM of the chimeric mice. As expected, the composition of the hematopoietic CD45^+^ cells in the BM reflected the initial injection ratio of 80% MD4/20% WT in both the WT and VM recipients (Figure 3B). However, the percentage of B cells derived from the BCR Tg donor in the BM, spleen, and EV lung dropped to ~20%–30%, as might be anticipated for cells with a restricted repertoire (Figure 3B).

Importantly, the total number of both WT and MD4-derived donor B cells in the EV lung was much higher in VM versus WT recipients, and WT B cells preferentially accumulated in VM lungs (Figure 3C). Together, these data show that the WT B cells generally outcompeted Tg MD4 B cells as far as accumulation in peripheral lymphoid compartments, although MD4 cells were not excluded from the lungs of VM recipients.

A large proportion of both the WT- and MD4-derived EV B cells (~50%) in the VM host were IgD^+^ naive B cells (Figure 3D and Supplemental Figure 1; supplemental material available online with this article; https://doi.org/10.1172/jci.insight.174331DS1). However, only WT donor cells in the lungs of the VM host became Fas^+^GL7^+^ GC B cells (Figure 3E). Thus, while most resident B cells in the VM lung are naive, a subset of B cells with diverse repertoires form GCs within the VM lung, potentially reflecting self-reactivity. We further analyzed our multiparameter flow cytometry data by dimensional reduction (Supplemental Figure 2A), unsupervised clustering, and manual cluster annotation of multiparameter flow cytometry data, as a complementary analytic approach to visualize broader patterns of B cell differentiation (Supplemental Figure 2B). The Uniform Manifold Approximation and Projection (UMAP) of our lung B cell data was organized such that UMAP1^hi^ (*x* axis) was associated with IgD, and UMAP2^hi^ (*y* axis) was associated with IgM and CD69 (Supplemental Figure 2C). We then arbitrarily identified B cell subsets by unsupervised clustering using a limited set of markers (Supplemental Table 1) and looked for subsets differentially represented across donor-host combinations. This analysis affirmed the presence of several activated B-lineage populations that were uniquely elevated in the WT→VM condition, including GC B cells (green oval) as well as plasma cells (PCs; black oval), and Fas^+^GL7^–^ B cells (FasB; red circle) (Supplemental Figure 2D and Figure 3, F and G). The repertoire-dependent enrichment for these subsets of activated B-lineage cells was not limited to the lung, as GC, PC, and FasB cells derived from WT donors were also found in the spleen of VM hosts (Figure 3, H–K). Collectively, we find that the VM lung effectively recruits both non-Tg and WT naive B cells that require a diverse repertoire to become further activated.

### Non-Tg T cells are preferentially recruited to and activated in the VM lung.

**Figure 4:**
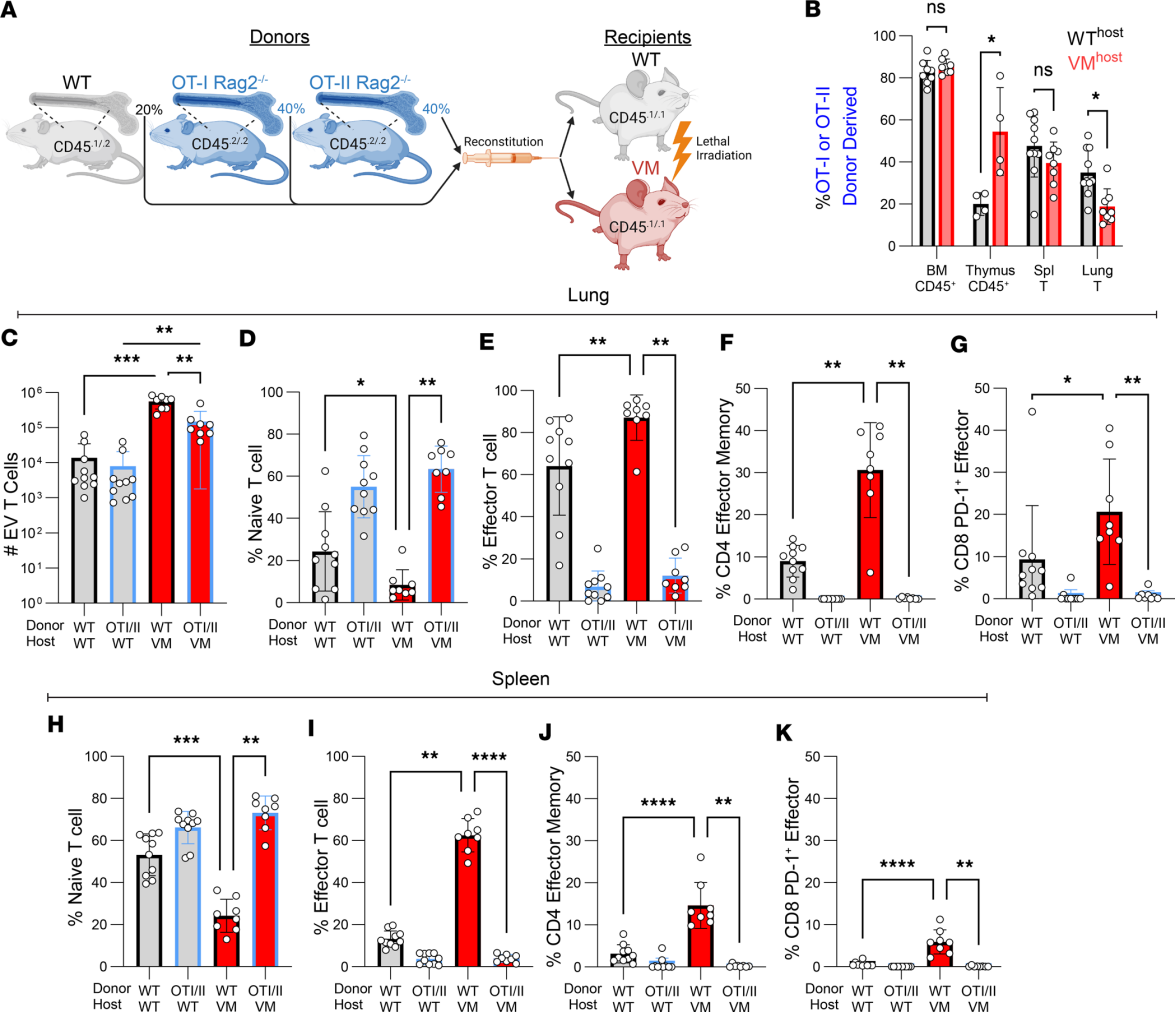
T lymphocyte recruitment and activation in VM ILD requires a diverse repertoire. (**A**) Six-week-old sex-matched CD45^.1/.1^ WT and VM littermates were lethally irradiated and reconstituted with a mixture of BM stem cells comprised of 20% WT CD45^.1/.2^, 40% OT-I Rag2^–/–^ CD45^.2/.2^, and 40% OT-II Rag2^–/–^ CD45^.2/.2^ donor BM. WT+OT-I/-II→WT (*n* = 4–10) and WT+OT-I/-II→VM (*n* = 4–8) chimeric mice were evaluated 8–9 weeks later. (**B**) Percentage of OT-I/-II donor-derived cells within the donor-derived CD45^+^ BM compartment, CD45^+^ thymus, CD3^+^TCRb^+^ splenic T cells, and CD3^+^ TCRb^+^ lung extravascular (EV) T cells. (**C**) Total number of lung EV T cells derived from either the WT or OT-I/-II donors in WT and VM hosts. (**D**–**G**) Percentage of CD44^–^CD62L^+^ naive T cells and percentage of CD44^+^CD62L^–^ effector T cells within lung EV T cells, percentage of CD127^+^CD69^–^ CD4 effector memory cells within CD4^+^ lung EV T cells, and percentage of PD-1^+^CD69^+^ CD8 PD-1 effector cells within CD8^+^ lung EV T cells derived from WT and OT-I/-II donors in WT and VM hosts. (**H**–**K**) Percentage of naive and effector T cells in splenic T cells, percentage of CD4 effector memory T cells within CD4^+^ splenic T cells, and percentage of CD8 PD-1 effector cells within CD8^+^ splenic T cells derived from WT and OT-I/-II donors in WT and VM hosts. For **C**–**K**, data points in columns 1 and 2 represent paired WT and OT-I/-II donors from a shared WT host. Similarly, data points in columns 3 and 4 represent paired WT and OT-I/-II donors from a shared VM host. To determine statistical significance, nonparametric Mann-Whitney *U* tests were used for pair-wise comparisons between columns 1 and 3 and between columns 2 and 4. A Wilcoxon matched pairs signed rank test was used for pairwise comparisons between columns 3 and 4. Statistical significance in **B** was determined using a multiple Mann-Whitney *U* test with FDR correction (*P < 0.05, **P < 0.01, ***P < 0.001, ****P < 0.0001).

TCRαβ T cell infiltration of the lung is a predominate feature of SAVI disease, and as with B cells, WT T cells are recruited and activated in the lungs of VM hosts (13). To determine whether a restricted TCR repertoire limits recruitment and/or activation of T cells to the SAVI lung, we utilized OT-I and OT-II Tg mice, which express TCRs specific for ovalbumin peptides bound to MHCI and MHCII, respectively. OT-I T cells become CD8 T cells, whereas OT-II T cells become CD4 T cells. Radiation chimeras were again used to assess the dependence of repertoire on T cell recruitment to the lungs in both CD4 and CD8 T cell subsets. To reconstitute both the CD4 and CD8 host compartments with repertoire restricted donors, lethally irradiated WT or VM hosts were given a mixture of Rag2^–/–^ OT-I (40%) and Rag2^–/–^ OT-II (40%) stem cells and allelically distinct non-Tg WT (20%) (2:2:1 ratio), indicated as OT+WT→WT and OT+WT→VM, respectively (Figure 4A). We then examined the BM, thymus, spleen, and lungs of chimeric mice 8 weeks later.

As in the BCR Tg experiments, the percentage of TCR Tg (OT-I + OT-II) donor-derived CD45^+^ BM cells reflected the donor cell inoculum (~80%) in both WT and VM hosts (Figure 4B). In contrast, the percentage of thymocytes derived from OT donor cells was reduced to ~20% in WT recipients. Intriguingly, the percentage of OT donor-derived thymocytes in the VM recipients increased to ~50% (Figure 4B). This difference could reflect defects in thymic education resulting from VM expression in thymic stromal cells (18). In the spleen, the frequency of Tg T cells in the VM hosts (~40%) was only mildly reduced compared with the WT hosts (~50%). However, in the VM lung EV T cell compartment, the frequency of Tg T cells was significantly reduced compared with WT host (Figure 4B).

Consistent with the increased number of EV T cells in VM mice, total numbers of OT and WT donor-derived T cells were significantly higher in the VM host (Figure 4C). However, in VM hosts, the number of OT donor-derived T cells was lower than the number of WT donor-derived T cells. In addition, most of the OT T cells in the lung were CD44^–^CD62L^+^ naive T cells in both the WT and VM hosts (~50%) (Figure 4D and Supplemental Figure 3). By comparison, CD44^+^CD62L^–^ effector subsets were almost entirely derived from the WT donor (Figure 4E), indicating that a diverse TCR repertoire favored T cell activation within the lung.

To expand upon our flow cytometric analysis on T cell activation using a complementary approach, we again utilized dimensional reduction (Supplemental Figure 4A), unsupervised clustering, and manual cluster annotation (Supplemental Figure 4B and Supplemental Table 2). Our UMAP was organized such that UMAP1^lo^ associated with CD4^+^ T cells, and UMAP1^hi^ associated with CD8^+^ T cells. UMAP2^lo^ associated with activated T cell markers CD44 and CD69 and UMAP2^hi^ associated with the naive T cell marker CD62L (Supplemental Figure 4C). Interestingly, while mice receiving WT donor BM showed a roughly expected 2:1 ratio of CD4/CD8 T cells, mice receiving OT Tg donor BM instead showed a roughly 1:1 ratio of CD4/CD8 T cells (Supplemental Figure 4D). Consistent with our conventional flow cytometric analysis above, we found that only WT donor T cells in both WT and VM host lung were mainly UMAP2^lo^, reflecting an enrichment for effector T cell subsets (Supplemental Figure 4D), whereas OT Tg T cells in the lungs of both WT and VM hosts were predominately UMAP2^hi^, reflective of naive and central memory T cells (Supplemental Figure 4D). Additionally, we identified clusters corresponding to effector memory CD4 T cells and PD-1^+^ effector CD8 T cells, as indicated by red and lime green circles in our UMAP (Supplemental Figure 4B), and observed their enrichment in WT→VM chimeras compared with WT→WT chimeras, as well as their near absence in OT donor T cells in either host (Supplemental Figure 4D and Figure 4, F and G). Thus, repertoire-restricted Tg T cells can be recruited to the lung where they retain their naive status despite substantial ongoing tissue inflammation. In contrast, repertoire unrestricted WT T cells are also recruited to the VM lung but further differentiate into a variety of activated populations. These activated T cell populations are not limited to the lung, as a repertoire-dependent enrichment of effector, CD4 effector memory, and CD8 PD-1^+^ effectors derived from WT donors was also observed in the spleen (Figure 4, H–K).

### WT→VM mice produce autoantibodies reactive against lung tissue and common autoantigens.

**Figure 5:**
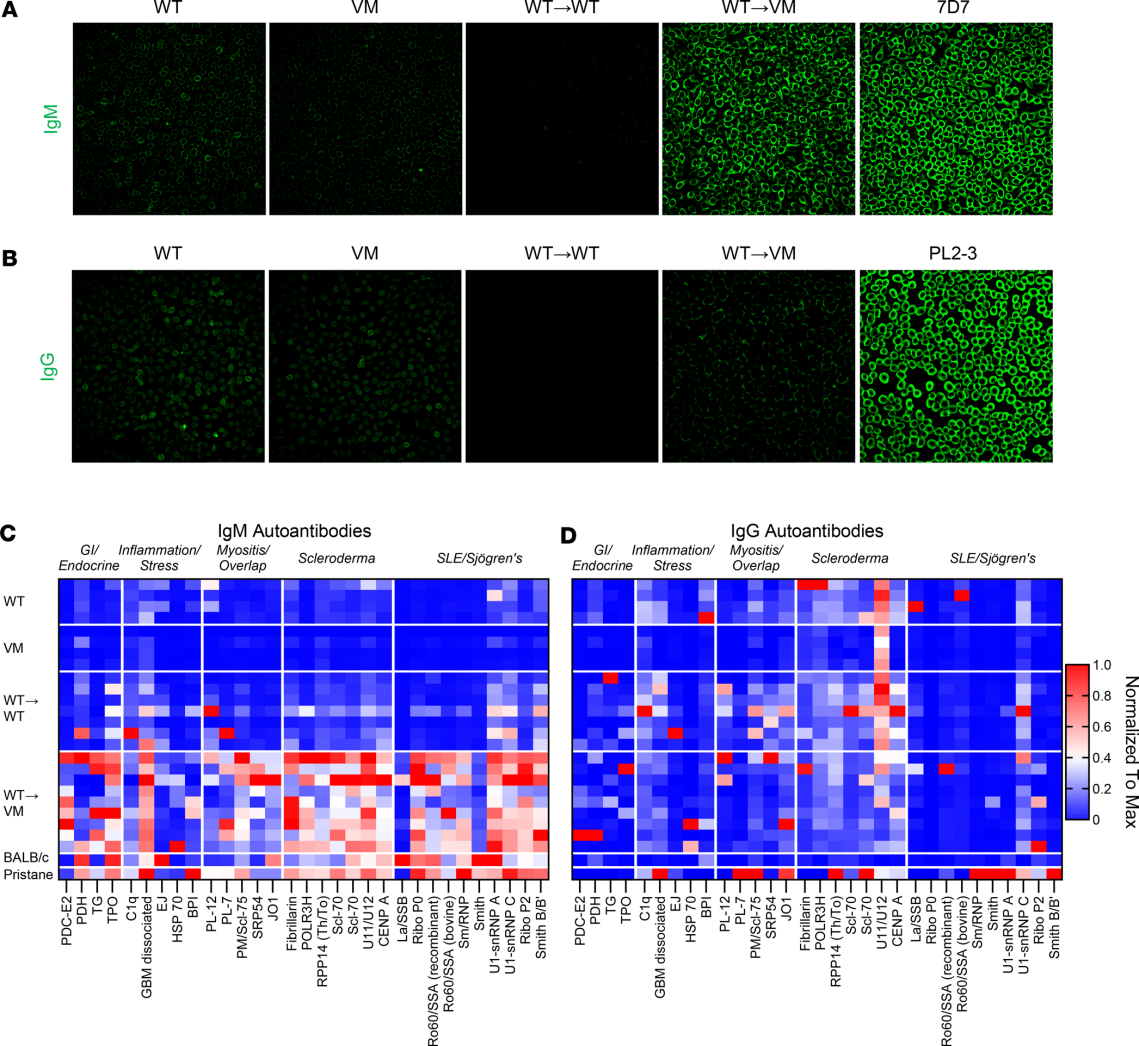
WT→VM mice produce IgM autoantibodies. (**A** and **B**) Immunofluorescence staining of HEp2 slides with representative sera of the indicated strains or chimeric mice with representative WT (*n* = 5) and VM (*n* = 3) mice as well as WT→WT (*n* = 16) and WT→VM (*n* = 17) chimeric mice. (**A**) Autoantibodies were detected with a Dylight 488–conjugated anti-IgM antibody (**A**) or anti-IgG antibody (**B**). 7D7 is an IgM anti-DNA antibody, and PL2-3 is an IgG_2a_ anti-chromatin antibody. Scale bars: 200 μm. (**C** and **D**) Sera from WT (*n* = 4), VM (*n* = 4), WT→WT (*n* = 7), WT→VM (*n* = 9), BALB/c (*n* = 1), and BALB/c mice treated with pristane (*n* = 1) were assessed by an autoantigen array as negative and positive reference controls. Data are column normalized to the highest and lowest signal observed for each autoantigen. Autoantigens are grouped according to the disease they are associated with: gastroenteric and endocrine autoimmunity (GI/endocrine), nonspecific inflammation and stress, myositis and overlap syndromes, scleroderma and systemic sclerosis, and SLE/Sjögren’s syndrome. **C** shows the reactivity of sera IgM, and **D** shows the reactivity of sera IgG.

The repertoire dependence of GC and PC formation in the VM lungs supported the notion that B cell differentiation in the VM lung depends on the recognition of self-antigen. To determine whether WT B cells activated in a VM lung make autoantibodies, we first screened for anti-nuclear antibodies (ANAs) using an immunofluorescence (IF) HEp2 staining assay (Figure 5, A and B). Remarkably, sera from WT→VM mice, but not the parental VM mice, made a robust IgM ANA; there was a more minimal IgG response. To further explore the specificity of these ANA-reactive antibodies, sera from WT, VM, and chimeric mice were screened on an autoantigen array. Consistent with the ANA staining patterns, only IgM antibodies from the WT→VM chimeras, and no other groups, showed extensive autoantigen reactivity, particularly against autoantigens common to scleroderma/systemic sclerosis and SLE/Sjögren’s syndrome (Figure 5, C and D, and Supplemental Figures 5 and 6).

**Figure 6:**
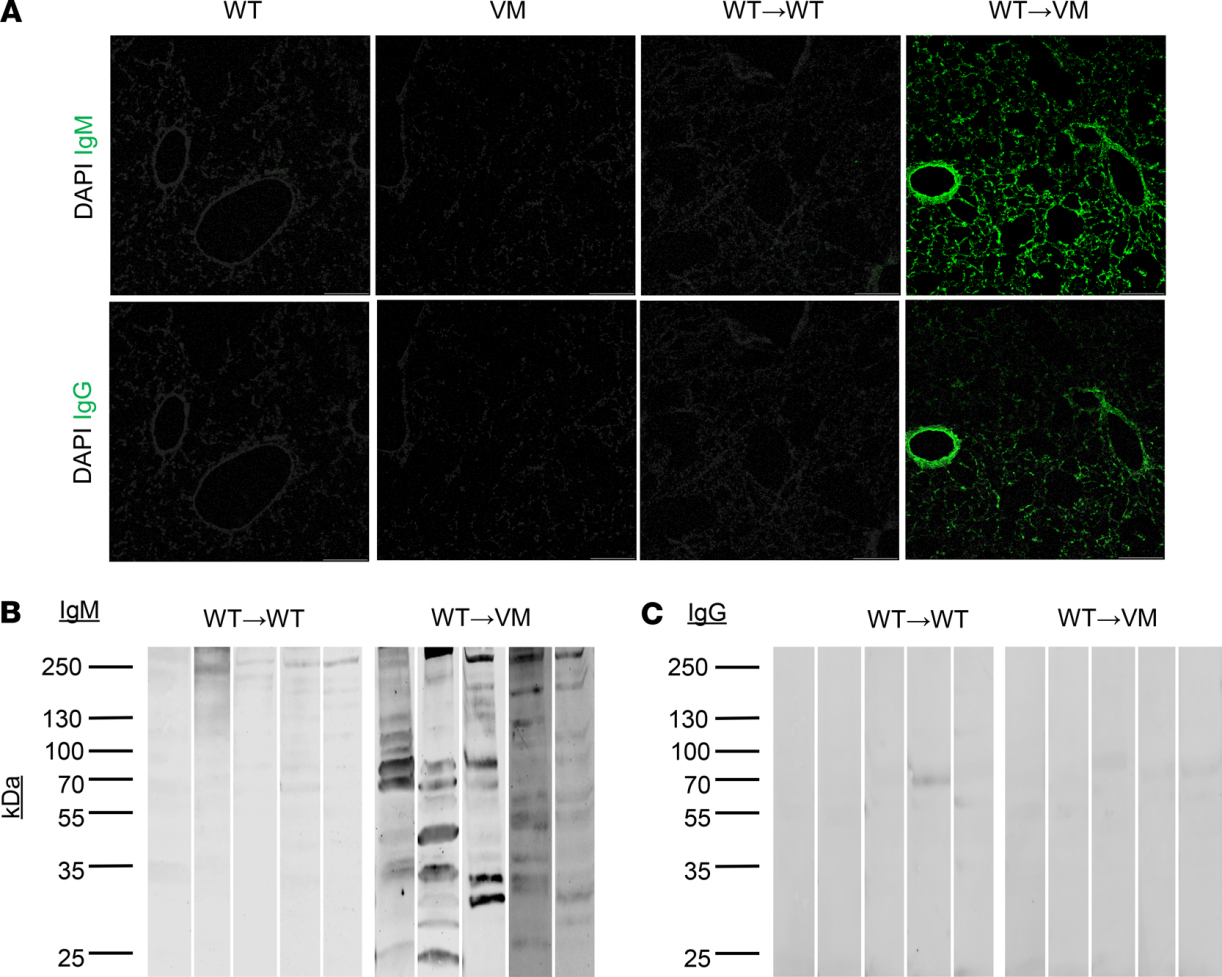
WT→VM autoantibodies target lung proteins. (**A**) Immunofluorescence staining of Rag1^–/–^ lung sections with serum samples diluted 1:40. A representative result is shown from WT (*n* = 3) and VM (*n* = 2) mice and from WT→WT (*n* = 10) and WT→VM (*n* = 10) chimeric mice. Slides were then developed with DAPI and Alexa Fluor 488–conjugated anti-IgG (top row) or anti-IgM (bottom row) antibody. Scale bars: 200 μm. (**B** and **C**) Lung reactive IgM and IgG antibodies were detected in sera from WT→WT or WT→VM chimeric mice (diluted 1:500) by Western blot against lysates from Rag1^–/–^ lungs. Numbers on the left side correspond to the mass in kDa of bands from the protein ladder. Data are representative of a total of *n* = 9 biologic replicates per group. The lanes in **B** and **C** were run on the same gel but were noncontiguous.

Given the sera autoantibody profile of WT→VM chimeras aligned with several systemic autoimmune diseases that frequently target the lung, we also screened sera for reactivity with lung tissue. We assessed direct binding to lung tissue by staining lung sections from Rag1^–/–^ mice with chimeric sera and by then detecting bound antibodies with fluorophore-conjugated anti–mouse IgM and IgG antibodies. IgM in the WT→VM sera clearly stained lung tissues, whereas IgG staining was more modest (Figure 6A). This may reflect a relatively higher abundance of IgM autoantibodies as compared with IgG autoantibodies in WT→VM chimeras. To confirm these findings using a complementary approach, sera from chimeric mice was used to stain lung lysates from Rag1^–/–^ mice by Western blot. As anticipated, IgM from WT→VM, but not WT→WT chimeras, detected a number of distinct protein bands, several of which were bound by more than 1 serum sample (Figure 6B). However, we did not detect marked reactivity of WT→VM IgG on lung lysates (Figure 6C).

In summary, even though VM mice produce very little circulating antibody and show minimal antibody reactivity against self-antigens, autoreactive WT B cells can be activated in VM hosts, differentiate into GC B cells, and produce autoantibodies directed against specific self-antigens and lung tissues.

### WT lymphocytes that develop in a VM host transfer ILD to a naive Rag1^–/–^ mouse.

**Figure 7:**
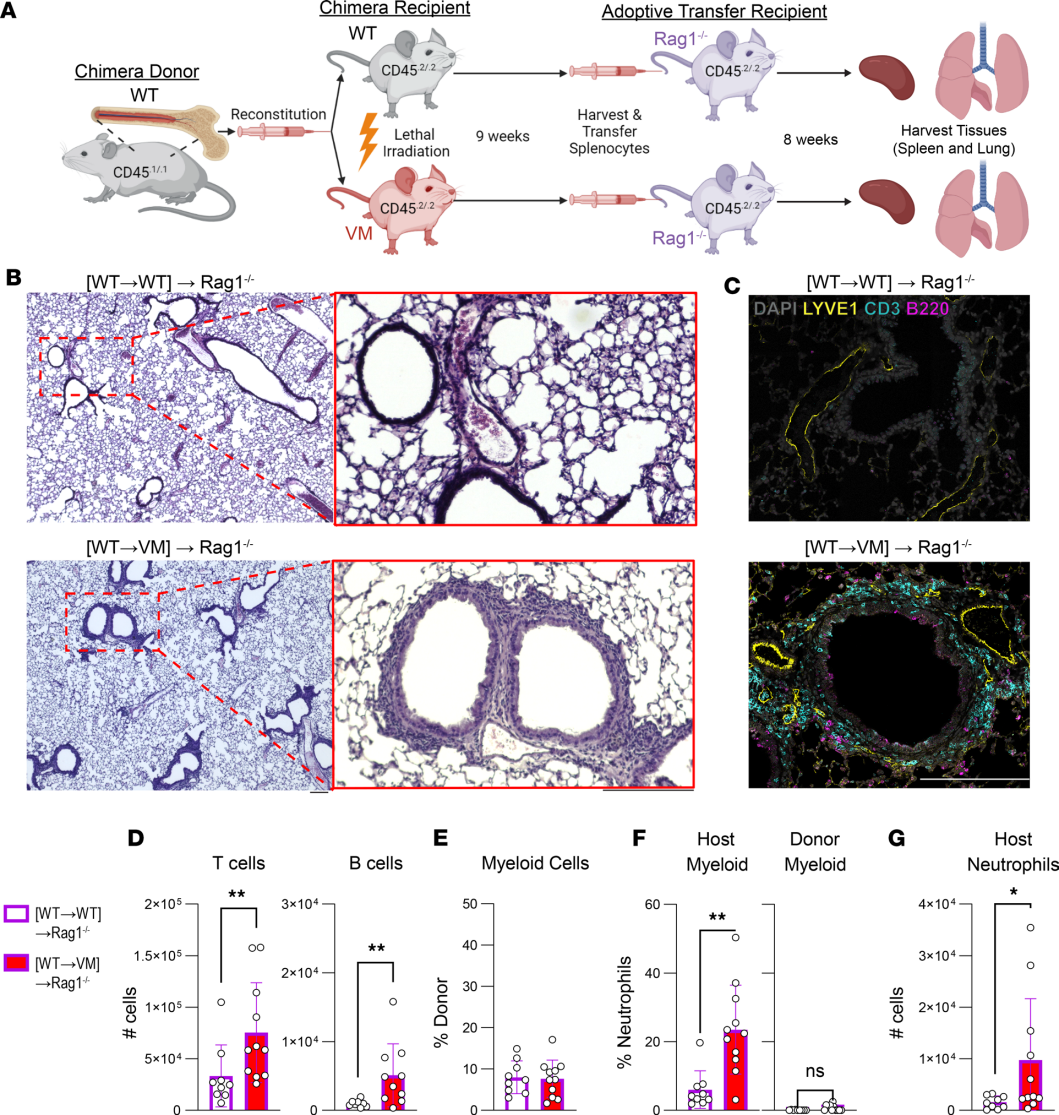
WT lymphocytes from WT→VM chimeras induce lung inflammation in Rag1-KO mice. (**A**) Six-week-old CD45^.2/.2^ WT and VM mice were lethally irradiated and reconstituted with CD45^.1/1^ WT donor BM to generate WT→WT and WT→VM chimeric mice. Splenocytes from these chimeric mice were then harvested 8 weeks after BM engraftment and adoptively transferred into secondary recipient 8-week-old Rag1^–/–^ recipients to generate (WT→WT)→Rag1^–/–^ (*n* = 9) and (WT→VM)→Rag1^–/–^ (*n* = 11) mice and then evaluated 8–9 weeks later. (**B**) Representative H&E lung histology from (WT→WT)→Rag1^–/–^ and (WT→VM)→Rag1^–/–^ mice captured with a 4× objective. A region of interest, as indicated by dotted red box, was further imaged with a 10× objective and shown 5× magnified over the original image. Data in **B** are representative of *n* = 9 (WT→VM)→Rag1^–/–^ mice and *n* = 11 (WT→WT)→Rag1^–/–^ mice. (**C**) Lungs from (WT→WT)→Rag1^–/–^ and (WT→VM)→Rag1^–/–^ mice, stained for DAPI (gray), CD3 (yellow), B220 (cyan), and LYVE-1 (magenta). Data in **C** are representative of *n* = 3 (WT→VM)→Rag1^–/–^ mice and *n* = 2 (WT→WT)→Rag1^–/–^ mice. (**D**) Total number of EV T and B cells in the lungs of (WT→WT)→Rag1^–/–^ and (WT→VM)→Rag1^–/–^ mice. (**E**) Percentage of donor-derived cells in the CD11b^+^ or CD11c^+^ lung EV myeloid compartment. (**F**) Percentage of CD11b^+^Ly6G^+^ neutrophils from the host and donor lung myeloid cell compartment. (**G**) Number of neutrophils from host lung EV myeloid compartment. Nonparametric Mann-Whitney *U* tests were used for pair-wise comparisons to determine statistical significance (*P < 0.05, **P < 0.01). Scale bar: 200 μm.

Another hallmark of autoimmunity is the ability to adoptively transfer autoreactive lymphocytes into naive mice and thereby transfer clinical manifestations of autoimmune disease (19). Since lymphocytes in WT→VM chimeras showed a robust and functional autoimmune phenotype with repertoire-dependent activation across both lung and spleen compartments (Figures 3 and 4), we decided to test chimeric splenocytes for their capacity to transfer lung inflammation to naive Rag1^–/–^ secondary recipient mice. Splenocytes were collected from WT→VM or WT→WT radiation chimeras at 9 weeks after reconstitution and injected into Rag1^–/–^ mice (Figure 7A). Eight weeks later, tissues were collected from the WT→WT and WT→VM secondary recipients. T and B cells from both donors engrafted the spleens of the secondary recipients (Supplemental Figure 7A); however, only recipients injected with splenocytes from WT→VM chimeras, but not WT→WT chimeras, developed modest, histologically apparent immune aggregates in the lung (Figure 7B). IF staining revealed the presence of a CD3^+^ peri-broncho-vascular T cell infiltrate (Figure 7C). Furthermore, flow cytometric analysis of lung tissue from mice that received WT→VM chimeric splenocytes affirmed increased numbers of EV T cells compared with lungs from WT→WT splenocyte recipients (Figure 7D).

There was also a modest increase in lung EV B cells in mice that received WT→VM chimeric splenocytes as compared with WT→WT chimeric splenocyte recipients; however, the total B lymphocyte cellularity in lungs was much lower than T cells, consistent with the minimal detection of lung-resident B cells by IF staining (Figure 7, C and D). Intriguingly, activation of T and B cell populations across both the spleen and lung did not differ between the 2 donors (Supplemental Figure 7, B and C), indicating that additional factors provided by VM expression within parenchymal and stromal tissues likely contribute to the lung-resident lymphocyte hyperactivation seen in the WT→VM chimeric lung.

We also found a small number of donor-derived myeloid cells in the lungs of all the secondary recipients (Figure 7E). However, increased percentages and numbers of neutrophils were found in the lungs of the mice that received splenocytes from WT→VM chimeras as compared with WT→WT chimera splenocytes (Figure 7, F and G). These neutrophils were all host derived (Figure 7F), as might be expected based on the low frequency of neutrophils in the adoptively transferred splenocyte population and the fact that neutrophils have a very short half-life. The presence of neutrophils in the lung was consistent with previous studies that demonstrate T cell–dependent recruitment of neutrophils to the lungs of VM mice (13). Overall, these data suggest that the WT→VM chimera spleens contains a population of lung homing autoreactive T cells that can promote lung inflammation.

## Discussion

Monogenic autoinflammatory diseases affecting NA sensing are frequently associated with autoantibody production (3, 4), and the development of autoantibodies is characteristic of murine models of monogenic inflammatory diseases such as Trex1 deficiency (20, 21) and DNase II deficiency (22). Patients with SAVI also commonly make autoantibodies, although mice expressing the SAVI STING mutation VM do not (5). We previously established that ILD in VM mice is driven by expression of the VM mutation in nonhematopoietic cells (13, 23). We now show that the inflammatory environment resulting from expression of the VM SAVI mutation by radioresistant cells is sufficient to promote the activation of autoreactive B and T cells as well as to promote the production of ANAs and antibodies reactive with proteins expressed in the lung. Thus, while lymphocyte-intrinsic STING activation constrains murine B cell activation and differentiation, VM-induced inflammation triggered by parenchymal/stromal cells in the lung promotes a loss of tolerance and the development of autoimmunity. Since human B cells do not express STING and, therefore, are not constrained by STING activation (12), WT→VM chimeras more closely recapitulate the development of autoantibodies as seen in patients with SAVI.

Our study builds upon prior work pointing to a role for autoreactive T cells in SAVI lung disease. OT-I mice crossed to mice expressing the STING mutation NS were found to have markedly reduced systemic and lung inflammation (9). The authors concluded that repertoire unrestricted CD8 T cells are required for SAVI ILD; however, while OT-I mice have normal numbers of CD8 cells, they are simultaneously deficient in CD4 T cells. Thus, the resulting phenotype of NS × OT-I mice cannot be solely attributed to a limited CD8 T cell repertoire and may also reflect the absence CD4 T cell effector populations or other pathogenic factors produced by CD4 T cell subsets. The mixed chimera approach utilized in this manuscript resolves this limitation and directly compares the recruitment and activation of repertoire-restricted CD4 and CD8 Tg T cells to repertoire-unrestricted WT cells, in the context of ongoing inflammation. We now demonstrate that T cell activation in VM SAVI mice requires a diverse repertoire, consistent with T cell autoreactivity.

We also find that a diverse repertoire is required for B cell activation in VM SAVI mice. This activation is associated with B cell autoreactivity, as WT→VM chimeric mice produce autoantibodies that directly bind to lung proteins and also recognize a range of autoantigens that are commonly targeted by B cells in systemic sclerosis and Sjögren’s syndrome, autoimmune diseases that commonly present with ILD (17, 24, 25). Similar autoantibody specificities have also been reported in patients with SAVI (e.g., SSA, RNP) (12, 26). Whether the autoantibody responses that develop in SAVI mice are protective (27) or pathogenic (13) remains to be determined and will be explored in future studies.

The exact specificity of these potentially autoreactive lymphocytes remains to be determined. We expect that it will be possible to detect oligoclonal expansion of self-reactive B and T lymphocytes through BCR/TCR repertoire sequencing studies. In addition, future studies utilizing mass spectrometry to identify proteins repeatedly detected by Western blot analysis of chimeric sera may help to identify specific proteins within lung tissues targeted by SAVI autoantibodies. These data may then be coupled to the identification of peptides that activate T cells present in the WT→VM chimeras. Furthermore, although the lung is a major target of SAVI pathology, patients with SAVI also present primarily with skin inflammation (5), and SAVI mice have also been found to develop spontaneous colitis (28). Whether these organ-specific pathologies also feature an autoimmune component remains to be determined.

Intriguingly, T cells activated in a WT→VM chimeric mouse retain the ability to home to lung tissues and promote neutrophil recruitment to the lung upon transfer to secondary unmanipulated naive host. It follows that inhibition of STING in patients with ongoing disease may not prevent continued pathology caused by existing autoreactive lymphocytes. Indeed, recent cases of lung disease recurrence have been observed in patients with SAVI who had undergone lung transplantation (29, 30). While this was potentially unexpected given our prior work demonstrating the role of radioresistant cell VM expression in SAVI disease (13), this unfortunate outcome may be explained by presence of autoreactive lung-targeting lymphocytes that persist in patients with SAVI and reinitiate lung pathology, as suggested by our study. Thus, therapeutic approaches to halt SAVI disease progression may need to mitigate both the innate immune activity of nonhematopoietic SAVI STING-expressing stroma/parenchymal cells and existing autoimmune lymphocytes for effective treatment of SAVI disease — for example, by sequential or combined BM and lung transplantation.

Collectively, our data support a multistep process wherein STING activation within nonhematopoietic lung cells promotes a repertoire-independent recruitment of immune cells into the lung environment and subsequent repertoire-dependent activation of lymphocytes results in the development of humoral autoimmunity directed against both lung tissues and systemic self-antigens. Our prior work suggests that endothelial cells may contribute to repertoire-independent recruitment of lymphocytes to the VM host lung, as endothelial cells exhibit a transcriptomic signature associated with chemokine production and as endothelial expression of the VM mutation is sufficient to cause pulmonary lymphocytic infiltration (13, 23). Our findings provide insight into how STING activation within stromal and parenchymal tissues initiates the development of autoimmunity and highlights the need to further investigate how engagement of cGAS-STING and other innate immune-sensing pathways within nonhematopoietic cells may contribute to pathogenesis of autoimmunity.

## Methods

### Sex as a biological variable.

Our study examined male and female animals, and similar findings are reported for both sexes.

### Mice.

STING VM SAVI mice were generated as described (6). SAVI VM mice and WT littermate controls were generated by crossing heterozygous male mice with WT females. MD4 (stock no. 002595) and B6 CD45.1 (stock no. 002014) mice were obtained from The Jackson Laboratory. MD4 mice were backcrossed to Rag1-KO mice (The Jackson Laboratory, 002216) to generate MD4 Rag1-KO mice. OT-I Rag2 KO (stock no. 2334) and OT-II Rag2 KO (stock no. 11490) mice were obtained from Taconic Biosciences. VM mice were also intercrossed with B6 CD45.1 mice to generate CD45 allotype–distinct hosts for chimera experiments. All mice used in these experiments were maintained in the same room and racks. STING^VM/WT^ (VM) and STING^WT/WT^ (WT) littermate and sex-matched controls were used for all experiments. Roughly equal numbers of male and female mice were used. Serum was collected by cardiac puncture of euthanized animals.

### Genotyping.

Rag1 genotypes were determined using DNA isolated from earpunches at the time of weaning. DNA was evaluated by PCR using the following primers 5′-GAG GTT CCG CTA CGA CTC TG-3′, 5′-CCG GAC AAG TTT TTC ATC GT, TGG ATG TGG AAT GTG TGC GAG-3′. Presence of the STING VM mutation was performed by Transnetyx.

### Generation of radiation chimeras.

Lethally irradiated (850R) 6- to 8-week-old mice were reconstituted with 1 × 10^7^ BM cells from sex- and age-matched mice. Mice were then placed on sulfatrim water and evaluated 8–9 weeks later.

### Splenocyte adoptive transfer.

Splenocyte adoptive transfer was performed as previously described (22). Single-cell suspensions were generated from donor spleens and RBC lysed (Sigma-Aldrich, R7757). In total, 30 × 10^6^ splenocytes were delivered into sex-matched 8-week-old naive Rag1-KO recipient mice. Engraftment of lymphocytes was confirmed 3 weeks later by analysis of blood. Mice were assessed 8–9 weeks after adoptive transfer.

### Intravascular labeling.

Circulating immune cells were identified as previously described (31). In brief, mice were injected i.v. with 3 μg of fluorescently labeled CD45 mAb and euthanized 3 minutes later. Lung was collected without subsequent perfusion and digested as described below.

### Lung digestion for hematopoietic cells.

To assess immune cells and endothelia from the left lobe of lung was digested using a GentleMACS lung digestion kit (Miltenyi Biotec, 130-095-927). In brief, lung was intratracheally inflated with 1 mL of GentleMACS lung digestion kit digestion buffer. Lungs were then incubated at 37°C and dissociated using a GentleMACS Octo Dissociator with Heaters (Miltenyi Biotec, 130-095-937). Cells were then filtered through a 70 μm mesh filter, spun down at 300*g* for 10 minutes, and treated with RBC lysis buffer (MilliporeSigma, R7757) before subsequent assessments.

### B cell magnetic bead purification.

The spleen was first dissociated in HBSS with 2% FBS between 2 frosted glass slides, pelleted by centrifugation at 300*g*, and RBC lysed. B220^+^ B cells were purified by magnetic particle selection (BD Biosciences 551513).

### B cell proliferation assays.

B cell proliferation was measured as previously described (32). Briefly, B cells were stained with VPD450 at 3.5 μM (BD Biosciences, 562158). Cells were stimulated with BLYS at 50 ng/mL and with an anti-IgM F(ab’)_2_ at 15μg/mL. On day 3, cells were stained with TO-PRO prior to flow cytometric assessment.

### Flow cytometry.

Cells were incubated in CD16/32 (Biocell, BE0307) and stained with antibodies as documented in Supplemental Tables 3–6. Samples were fixed using Fluorofix buffer (BioLegend, 422101). Intracellular staining was performed using a BD Biosciences Fixation/Permeabilization kit (BD Biosciences, 554714) after stimulating cells for 4 hours with Brefeldin A (BioLegend, 420601). Absolute cell counts were determined using counting beads (BioLegend, 424902). Cells were acquired on an Aurora (Cytek) or a ZE-5 (Bio-Rad) cytometer and analyzed with FlowJo software.

### Unsupervised machine learning analysis of flow cytometric data.

To analyze high-parameter flow cytometry data, we employed analysis by unsupervised machine learning tools. In brief, samples were annotated with metadata to identify individuals within cohorts. Gating was performed to identify lineage-positive populations across specific organ compartments (EV lung), and these populations were concatenated into a single FCS file. UMAP dimensional reduction was performed using select parameters for visualization. Unsupervised clustering was performed on the same parameters used for UMAP dimensional reduction using the Phenograph algorithm, since prior studies indicated that Phenograph showed high robustness with larger data sets (33). Metadata annotations were then used to deconvolve concatenated data and resegregate events into experimental and control groups. Cluster distributions for individuals within cohorts were calculated, and multiple-comparison testing was performed to identify differentially represented clusters across experimental conditions. The identity of clusters was determined through manual annotation using ClusterExplorer, conventional gating, and multiparameter heatmap analysis. Analysis was performed in FlowJo 10 and GraphPad Prism 9.

### Immunoglobulin analysis.

ELISAs for IgM, IgG1, and IgG2a were measured using a previously described sandwich ELISA (34). Polyclonal goat anti-IgM (Southern Biotech, 1020-01), anti-IgG1 (BD Biosciences, 557273), and anti-IgG2a (Southern Biotech, 1077-01) capture antibodies were coated onto plates at 1 µg/mL. A biotinylated-conjugated anti-κ light chain antibody (BD Biosciences, 559750) and streptavidin-HRP (Southern Biotech, 1050-05) were used for detection.

### Histology.

Lungs were dissected, inflated intratracheally with 10% phosphate-buffered formalin (PBF) via a flexible catheter, fixed in 10% PBF at room temperature for 48 hours, and transferred into 70% ETOH. Lungs were then paraffin embedded, sectioned, and stained with H&E by Applied Pathology Systems. Whole H&E lung slides were scanned at 4× or 10× using an EVOS FL Auto microscope or an EVOS M7000 microscope housed in the Bone Analysis Core (University of Massachusetts Chan Medical School).

### Immunofluorescence.

Sections were generated from either formalin-fixed, paraffin-embedded (FFPE) or fresh-frozen lungs (OCT). For FFPE sections, 7 μm–thick sections were prepared from FFPE blocks by the University of Massachusetts Chan Medical School Morphology Core. After deparaffinization using xylene, antigen retrieval was performed with 10 mM Na citrate 0.05% (MilliporeSigma) Tween 20 (MilliporeSigma) in a pressure cooker for 15 minutes. For OCT lung sections, lungs were inflated with a 1 mL of a 1:1 PBS/OCT, tied off at the trachea with surgical suture, and snap frozen in a bath of dry ice chilled 70% ETOH. Sections 7–40 μm thick were fixed in acetone for 10 minutes at –20°C. FFPE or OCT sections were then permeabilized in 0.3% Triton X-100, blocked in 10% donkey sera, incubated with primary antibody overnight at 4°C, and incubated with secondary antibody for 1 hour at room temperature. Antibodies are listed in Supplemental Tables 7 and 8. Microscopy was captured on a Leica SP8 confocal microscope or a Leica Thunder widefield fluorescence microscope and analyzed in the Leica Application Suite X.

### ANA assay.

Sera diluted 1:25 in 0.2% BSA PBS were evaluated by immunofluorescence microscopy using HEp-2 slides (Bio-Rad, 26102). mAbs PL2-3 (IgG2a) and 7D7 (IgM) were used as positive controls (2 mg/mL). Bound antibodies were detected with goat anti–mouse IgG-Dylight 488 (Invitrogen, A-11001) or goat anti–mouse IgM-Dylight 488 (Invitrogen, SA5-10150).

### Autoantigen array.

Samples were evaluated on autoantigen arrays described previously (35). Mouse sera were diluted 1:100 in an assay buffer of 0.05% PBS-T supplemented with 3% (w/v) BSA (MilliporeSigma) and transferred into a 96-well plate. Negative assay control included BALB/cJ WT mouse serum, and positive assay control included mouse serum from a pristane model of SLE as previously published (36). The bead array was distributed into a 384-well plate (Greiner BioOne) by transfer of 5 μL bead array per well. In total, 45 μL of the 1:100 diluted sera were aliquoted and transferred into the 384-well plate. Samples were incubated for 90 minutes on a shaker (Grant Bio) at RT. The beads were washed 3× with 60 μL PBS-T on a plate washer (EL406, BioTek). Secondary antibodies, including 50 μL of R-PE conjugated Fcγ fragment–specific goat anti–mouse IgG (Jackson ImmunoResearch, 115-115-071) and R-PE conjugated μ chain–specific goat anti–mouse IgG (Jackson ImmunoResearch, 115-116-075), were diluted 1:500 and 1:400, respectively, in 3% BSA in 0.05% PBS-T based on optimized assay conditions and transferred to the 2 halves of the 384-well plate, allowing for a parallel detection of different isotypes. After incubation with the secondary antibody for 45 minutes, the plates were washed 3× with 60 μL PBS-T and resuspended in 50 μL PBS-T for readout in a FlexMap3D instrument (Luminex Corp).

Data analysis and visualization was performed using R Studio (2023.03.0) and GraphPad Prism 9.5.1. Data were normalized by subtracting MFI values of unconjugated bare beads from antigen-coupled beads for each sample (37). The normalized data sets were used to generate bar graphs in R Studio using the “ggplot2” package (Supplemental Figures 5 and 6), while heatmaps were generated with Prism (Figure 5).

### Lung lysate Western blots.

Lungs were dissected from Rag1-KO mice following cardiac perfusion with 7 mL of ice-cold PBS, followed by 3 intratracheal washes with 1 mL of ice-cold PBS, and placed into M Tubes (Miltenyi Biotec, 130-093-236) with 6 mL of RIPA Lysis buffer (Thermo Fisher Scientific, 89900) with 1× HALT protease inhibitor cocktail (Thermo Fisher Scientific, 78429). Lungs were disassociated with a gentleMACS octodissociator using the gentleMACS Program Protein_01. Samples were then incubated on ice for 30 minutes and then sonicated for 2 minutes using 10-second sonication, 10-second rest cycles. Samples were then centrifuged at 4,000*g* for 20 minutes at 4°C. Lysate protein concentration was quantified using a DC assay (Bio-Rad, 3000111). Nupage reducing buffer and loading buffer were added to lysates prior to boiling samples at 95°C for 5 minutes. In total, 30 μg of lysate protein was loaded into wells of a Mini-Protean Precast gels (Bio-Rad, 4568024) alongside PageRuler Plus stained protein ladder (Thermo Fisher Scientific, 26619). SDS-PAGE was then performed at 120 V for 1 hour. Protein was then transferred onto a nitrocellulose membrane using semidry transfer run at 110 mA for 80 minutes. Membranes were then blocked with 5% nonfat dry milk in 0.1% TBS-Tween 20. Membranes were then stained with 1:500 sera diluted in blocking buffer overnight at 4°C with shaking. After washing off sera, secondary antibody cocktail was added containing 1:20,000 goat anti–mouse IgG IRDye 680RD (LI-COR, 926-68070) and 1:20,000 goat anti–mouse IgM IRDye 800CW (LICOR, 926-32280). Membranes were then visualized using a LI-COR Odyssey Imager and analyzed in Image Studio Lite V5.2.

### Statistics.

Data are represented as mean ± SD in our figures. Given that the number of biologic replicates in this study was *n* < 15, we chose to use nonparametric tests to remove the assumption that our data were normally distributed with statistical testing. For single comparisons, Mann-Whitney *U* tests were performed. For multiple-comparison testing, multiple–Mann-Whitney *U* tests were used with FDR correction using the 2-stage step-up method of Benjamini, Krieger, and Yekutieli. For paired testing comparing distinct donor populations within a shared host as featured in Figures 3 and 4, a Wilcoxon matched pairs signed rank test was used. A *P* value of less than 0.05 was considered significant.

### Study approval.

All animal experiments were conducted in accordance with the IACUC at the University of Massachusetts Chan Medical School.

### Data availability.

Values for all data points in graphs are reported in the Supporting Data Values file. New analytic code was not generated during this study.

## Author contributions

Conceptualization was performed by KMG, KN, AMR, and KAF. Methodology and experimental design were developed by KMG, KC, KN, AMR, KAF, XY, and PJU. Investigation and conducting of experiments were performed by KMG, KC, KN, SS, and XY. Experimental validation was performed by KC and SS. Formal analysis of data was performed by KMG and XY (Supplemental Figures 5 and 6). XY and PJU provided the autoantibody specificity assay. Writing of the original draft was performed by KMG. Subsequent review and editing was performed by KMG, AMR, KAF, and KN. Visualization of the data and preparation of figures was performed by KMG and XY (Supplemental Figures 5 and 6). Supervision was provided by AMR, KAF, and KN. All authors reviewed and commented on this manuscript.

## Supplementary Material

Supplemental data

Unedited blot and gel images

Supporting data values

## Version 1 07/18/2024

In-Press Preview

## Version 2 08/22/2024

Electronic publication
