# Supplementary material for: Activation of autoreactive lymphocytes in the lung by radioresistant cells expressing a STING gain-of-function mutation
Source: JCI Insight. 2024 Jul 18;9(16):e174331. doi: 10.1172/jci.insight.174331 (PMC11343592; doi:10.1172/jci.insight.174331)
Supplement: Supplemental data [file jciinsight-9-174331-s008.pdf]

Supplemental PDF

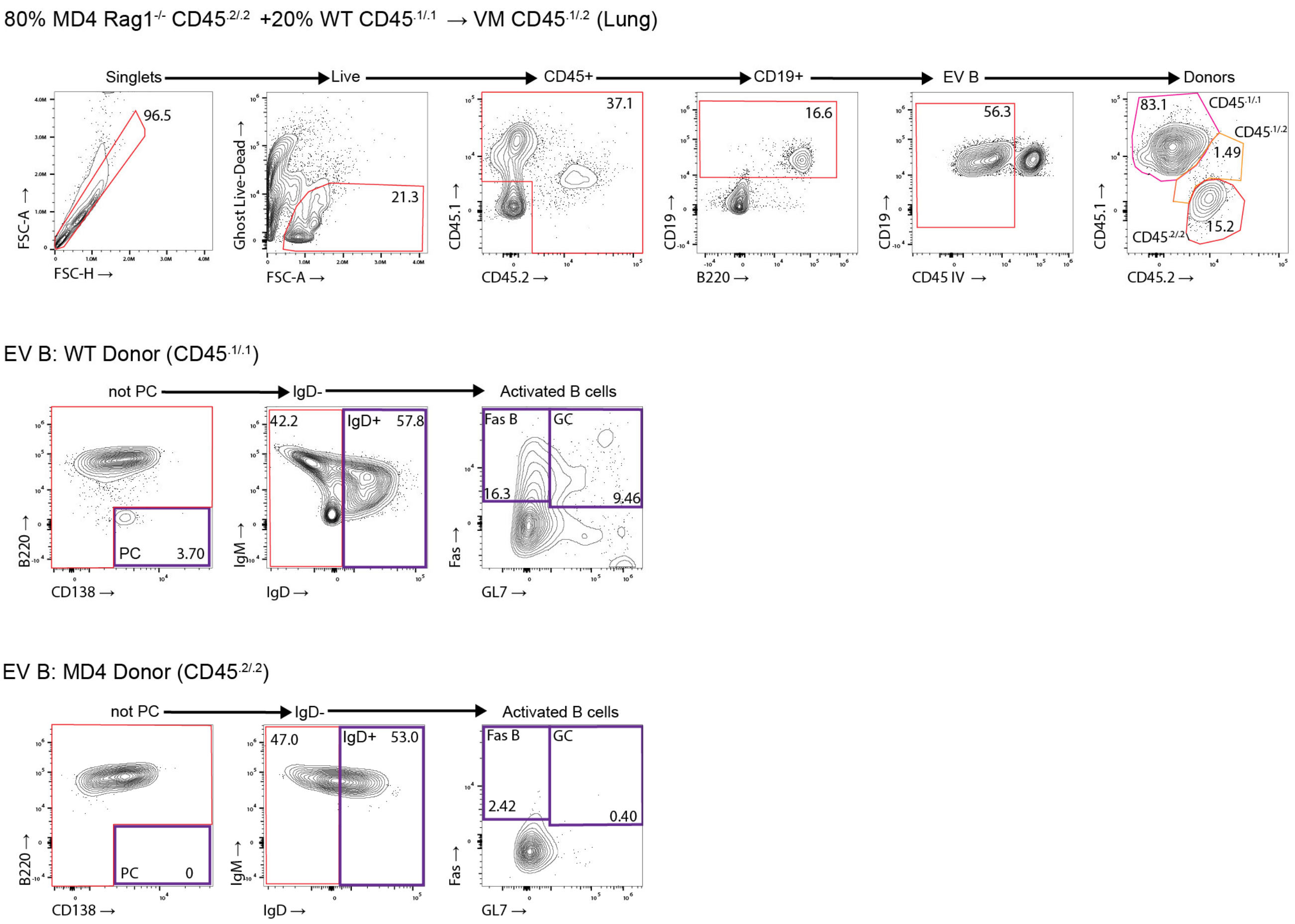

Supplemental Figure 1. Gating strategy for lung resident B cells in WT+MD4 mixed chimeras

Example flow cytometry from a VM CD45$^{.1/.2}$ mouse irradiated and reconstituted with 80% MD4 Rag1$^{-/-}$ CD45$^{.2/.2}$ and 20% WT CD45$^{.1/.1}$ bone marrow. Text above each bivariate plot describes the gate highlighted in red. Arrows indicate that this population was then sequentially gated on by the indicated markers in the adjacent right plot. The second and third row then stratify the analysis by either WT or MD4 donor status. Gates of populations shown in Figure 3 are highlighted in purple. Extravascular (EV); Plasma Cell (PC), Germinal Center (GC).

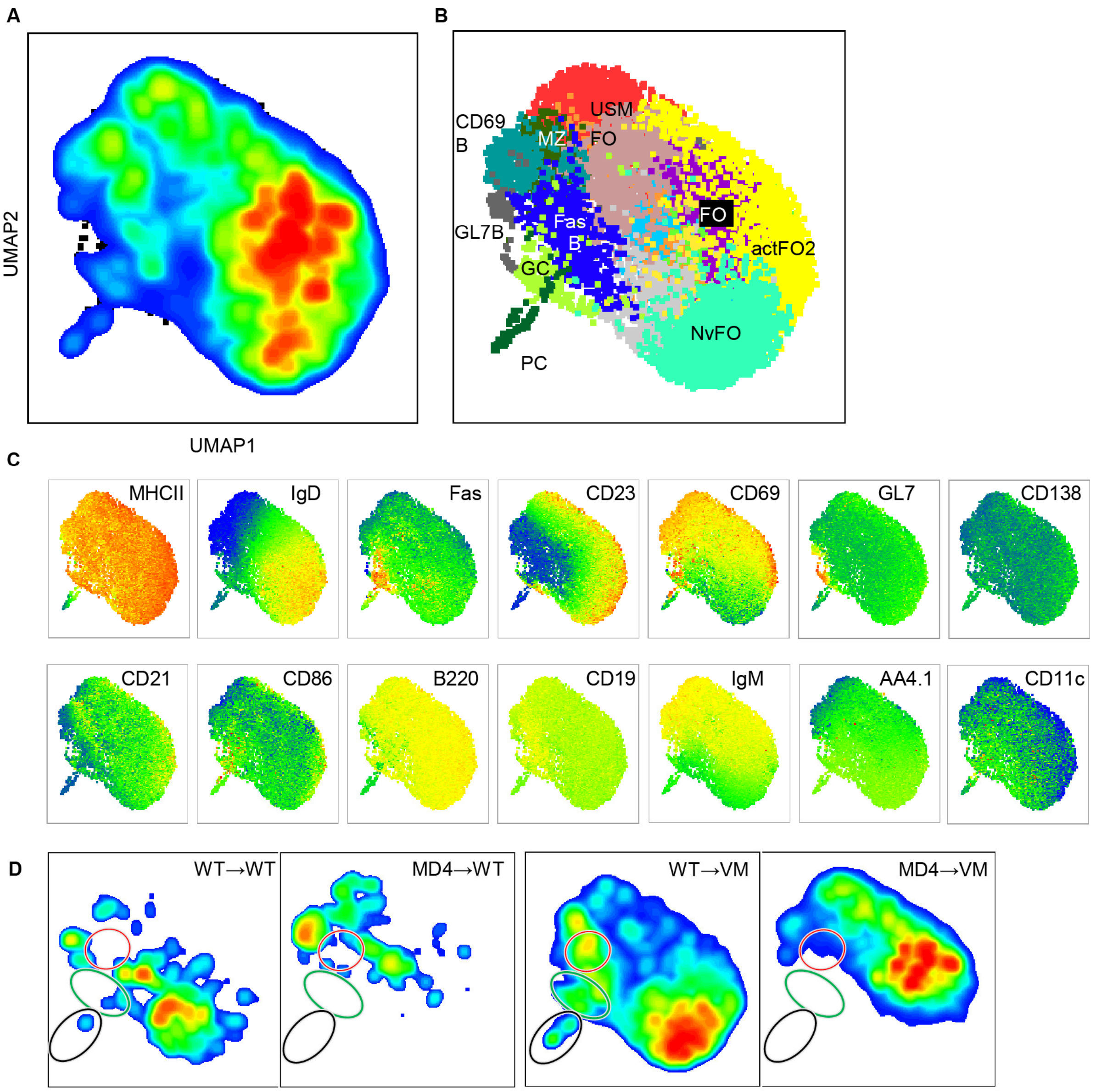

Supplemental Figure 2. Unsupervised clustering of B cell markers identifies repertoire and VM host dependent phenotypes.

1000 Live $CD45^{+}CD45IV^{neg}CD19^{+}$ lung extravascular (EV) B cell flow cytometry events per mouse from WT+MD4→WT (n=4) and WT+MD4→VM (n=6) mice were concatenated after excluding CD45.1 single positive radioresistant events. (**A**) A UMAP was generated using MHCII, IgD, Fas, CD23, CD69, GL7, CD138, CD21, CD86, B220, CD19, IgM, AA4.1, and CD11c. (**B**) Unsupervised clustering using Phenograph identified 10 clusters in this dataset, which were manually annotated based on their expression profile (Supplemental Table 1) and projected onto the UMAP alongside their annotated name. (**C**) shows heatmap projections for each parameter used to generate the UMAP and perform unsupervised clustering. (**D**) UMAPs depicting events from WT donor-derived cells in either WT (WT→WT) or VM (WT→VM) hosts, as well as events from MD4 donor-derived cells in either WT (MD4→WT) or VM (MD4→WT) hosts. Regions of the UMAP enriched in WT→VM

events are highlighted with rings corresponding to the Fas^+ cluster (red), the germinal center (GC) cluster (green), and the plasma cell (PC) cluster (black).

Markers	Name
B220+CD138-MHCII+	
IgD+	
CD21+CD23+	
CD69-CD86-	NvFO
CD69+CD86+	actFO2
CD69+CD86-	actFO1
IgD-	
IgM+CD21+CD23+	USM FO
IgM+CD21+CD23-	MZ
CD21-CD23-	
Fas+GL7-	FasB
Fas-GL7+	GL7B
Fas-CD69+	CD69B
Fas+GL7+	GC
B220-CD138+MHCII-	PC

Supplemental Table 1. Annotation of B cell clusters by marker expression

Manual annotation of 10 clusters identified by Phenograph unsupervised clustering of Live $CD45^{+}CD45IV^{neg}CD19^{+}$ lung extravascular (EV) B cell flow cytometry events from WT+MD4→WT (n=4) and WT+MD4→VM (n=6) mice concatenated after excluding CD45.1 single positive radioresistant events. Nested markers that describe each cluster are shown in the left column; whereas, given names for the clusters are shown in the right column.

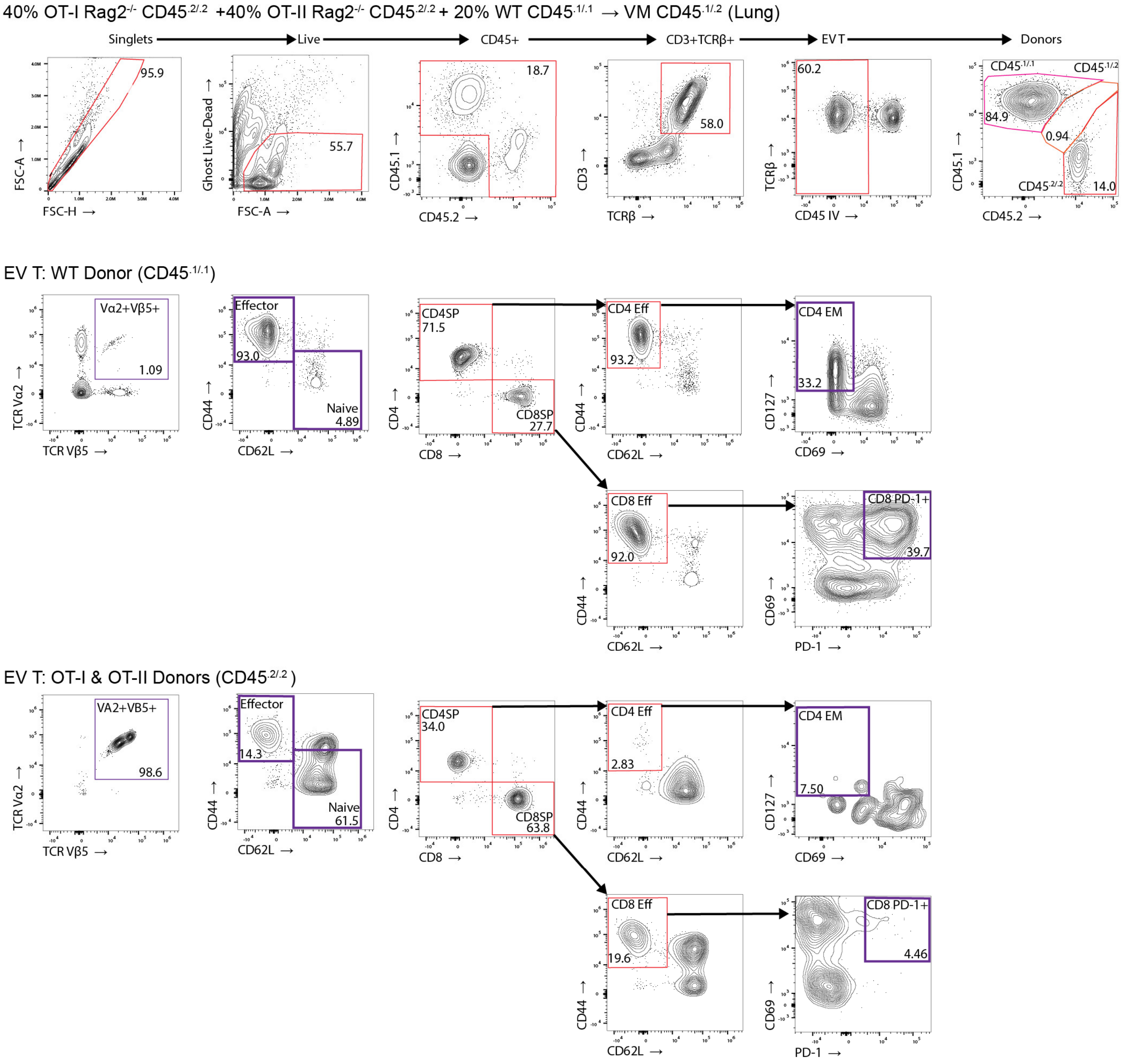

Supplemental Figure 3. Gating strategy for lung resident T cells in WT+OTI/II mixed chimeras

Example flow cytometry from a VM CD45.1/.2 mouse irradiated and reconstituted with 40% OT-I Rag2-/- CD45.2/.2, 40% OT-II Rag2-/- CD45.2/.2,and 20% WT CD45.1/.1 bone marrow. Text above each bivariate plot describes the gate highlighted in red. Arrows indicate that this population was then sequentially gated on by the indicated markers in the adjacent right plot. The second and third row then stratify the analysis by either WT or OT-I/II donor status. Gates of populations shown in Figure 4 are highlighted in purple. Extravascular (EV); Effector (Eff); Effector Memory (EM).

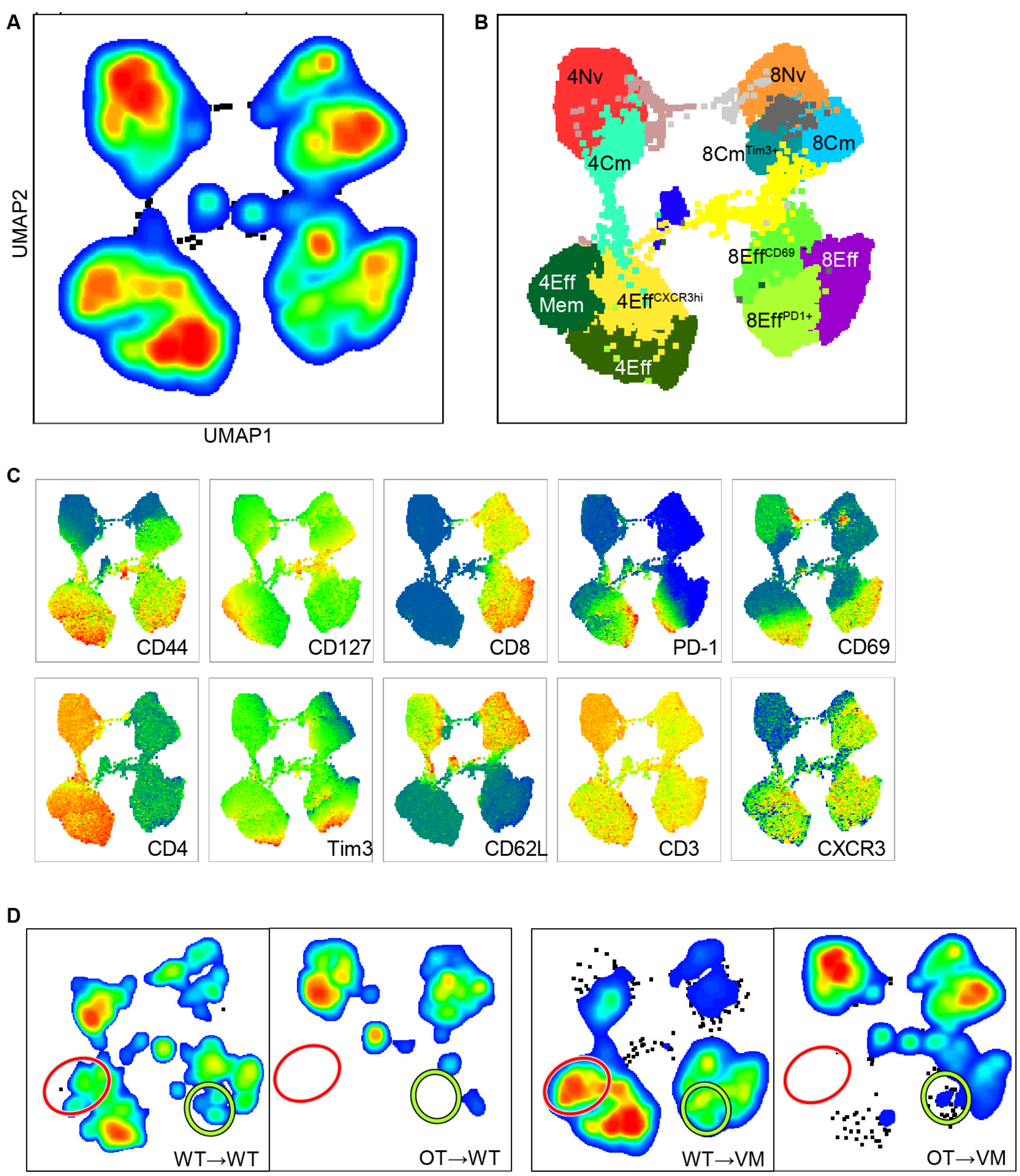

Supplemental Figure 4. Unsupervised clustering of T cell markers identifies repertoire and VM host dependent phenotypes.

2000 Live CD45+ CD3+ TCRβ+ lung extravascular (EV) αβ T cell flow cytometry events per mouse from WT+OTI + OTII→WT (n=5) and WT+ OTI + OTII→VM (n=5) mice were concatenated after excluding CD45.1 single positive radioresistant events. (**A**) A UMAP was generated using CD44, CD127, CD8, PD-1, CD69, CD4, Tim3, CD62L, CD3, and CXCR3. (**B**) Unsupervised clustering using Phenograph identified 12 clusters in this dataset, which were manually annotated based on their expression profile (Supplemental Table 2) and projected onto the UMAP alongside their annotated name. (**C**) Heatmap projections for each parameter used to generate the UMAP and perform unsupervised clustering. (**D**) UMAPs depicting events from WT donor-derived cells in either WT (WT→WT) or VM (WT→VM) hosts, as well as events from OTI/II donor-derived cells in either WT (OT→WT) or VM (OT→WT) hosts. Regions of the UMAP enriched in WT→VM events are highlighted

with rings corresponding to the CD4 effector memory (CD4 Eff Mem; 4Eff Mem) cluster (red), and the CD8 effector PD-1 (CD8 Eff PD1; 8Eff PD1^{+}) cluster (lime-green).

Markers	Name
CD3+TCRb+	
CD4+	
CD44-CD62L+	4Nv
CD44+CD62L+	4Cm
CD44+CD62L-	
CD127+CD69-CXCR3-PD1-	4Eff Mem
CD127-CD69-CXCR3+PD1+	$4Eff^{CXCR3hi}$
CD127-CD69+CXCR3+PD1+	4Eff
CD8+	
CD44-CD62L+	8Nv
CD44+CD62L+	
Tim3+	$8Cm^{Tim3+}$
Tim3-	8Cm
CD44+CD62L-	
CD69+PD1+	$8Eff^{PD1+}$
CD69+PD1-	8Eff
CD69-PD1-	$8Eff^{CD69-}$

Supplemental Table 2. Annotation of T cell clusters by marker expression

Manual annotation of 12 clusters identified by Phenograph unsupervised clustering of Live CD45+CD45IV-CD3+TCRβ+ lung extravascular (EV) αβ T cell flow cytometry events from WT+OTI/II→WT (n=5) and WT+OTI/II→VM (n=5) mice concatenated after excluding CD45.1 single positive radioresistant events. Nested markers that describe each cluster are shown in the left column; whereas, given names for the clusters are shown in the right column.

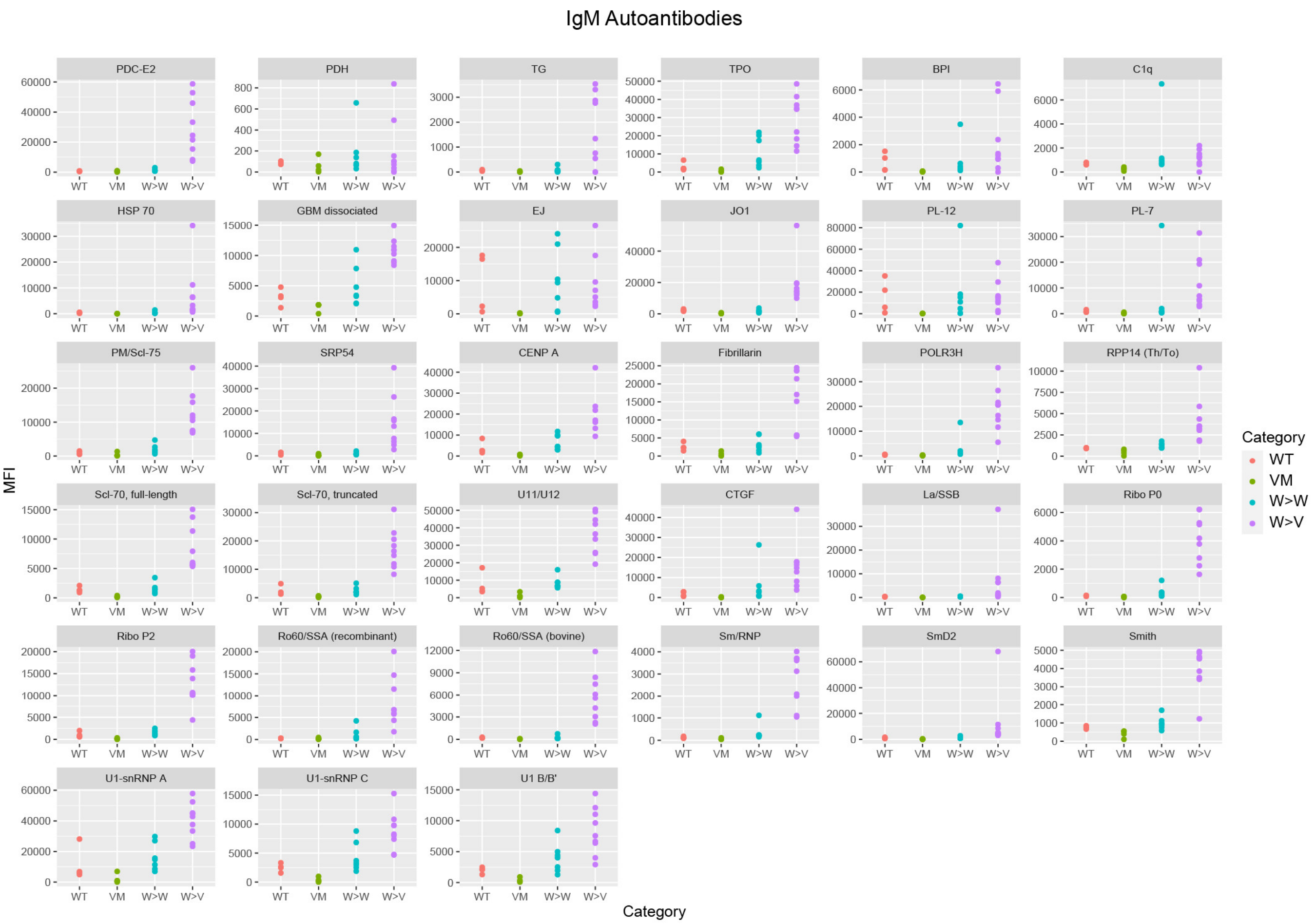

Supplemental Figure 5. IgM Autoantigen Array

Autoantigen mean fluorescence intensity (MFI) of mouse sera IgG antibodies from WT (n=4), VM (n=4), WT→WT (n=7), WT→VM (n=9), BALB/c (n=1), and BALB/c mice treated with pristane (n=1) as detected using an anti-mouse IgG detecting antibody.

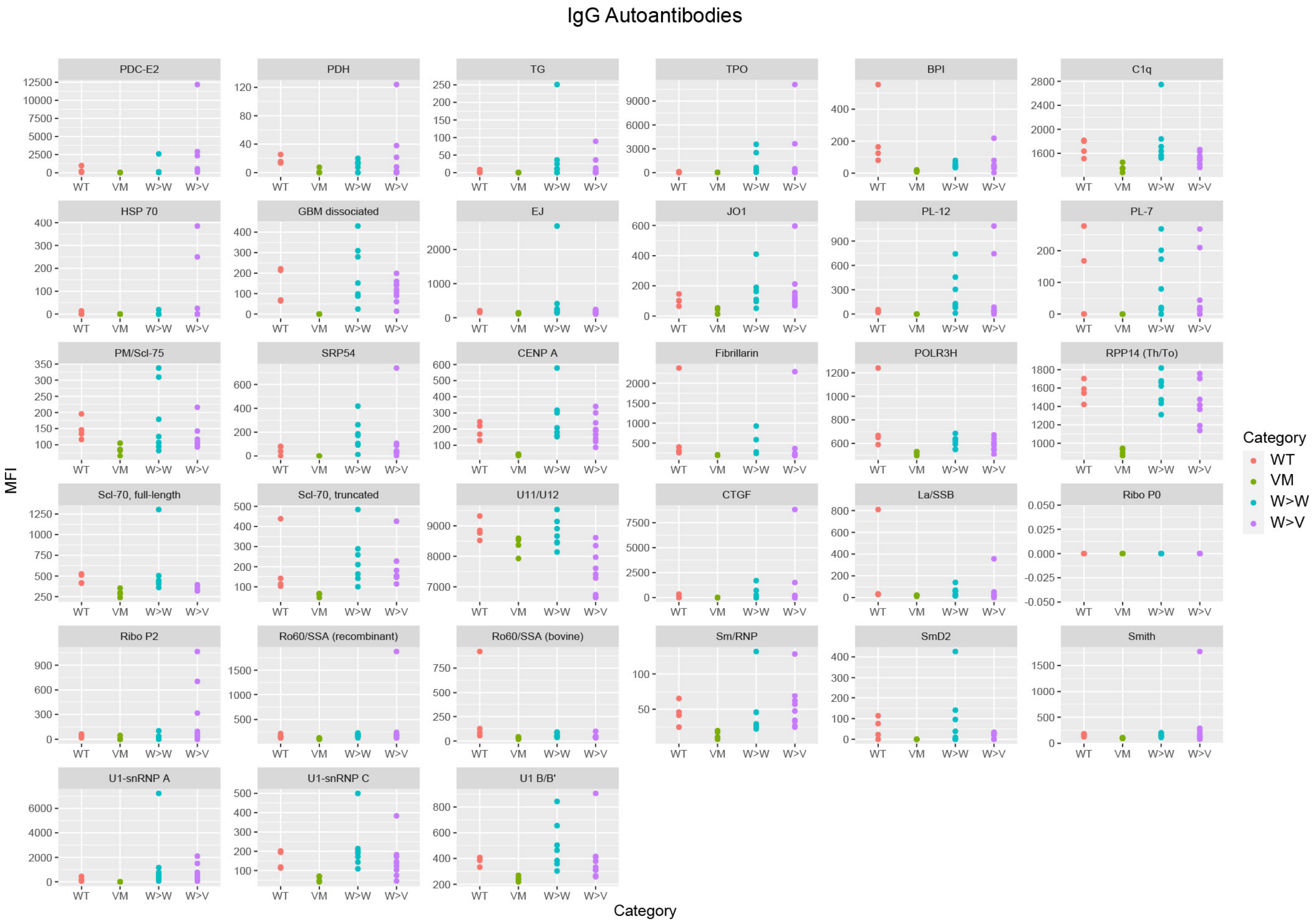

Supplemental Figure 6. IgG Autoantigen Array

Autoantigen mean fluorescence intensity (MFI) of mouse sera IgG antibodies from WT (n=4), VM (n=4), WT→WT (n=7), WT→VM (n=9), BALB/c (n=1), and BALB/c mice treated with pristane (n=1) as detected using an anti-mouse IgG detecting antibody.

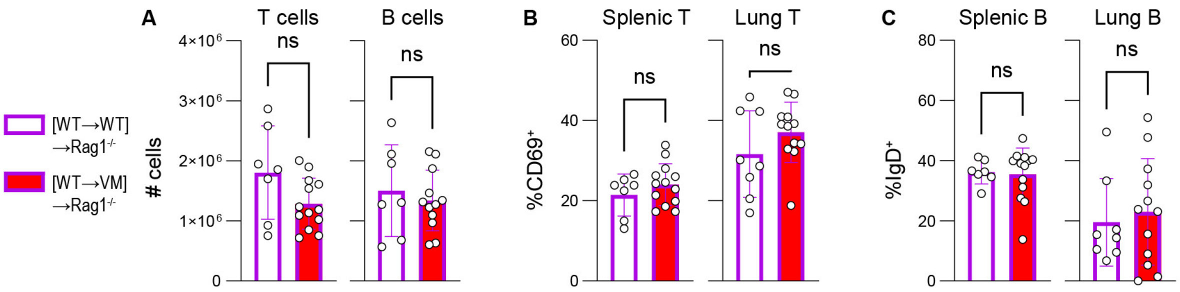

Supplemental Figure 7. WT lymphocytes from WT→VM chimeras engraft the spleen but do not show signs of increased activation.

Cohorts described in Figure 7 were assessed for (**A**) the total number of $CD3^+$ splenic T cells and $CD19^+$ splenic B cells. (**B**) $\%CD69^+$ in T cells from the spleen and resident within the lung. (**C**) $\%IgD^+$ in B cells from the spleen and resident within the lung. Nonparametric Mann-Whitney U-tests were used for pair-wise comparisons to determine statistical significance, (ns $p>0.05$).

Supplemental Table 3 Pan-immune flow cytometry antibody panels

Pan-Immune Panels

Figures	Reagent	Source	Catalog #	RRID
3B, 4B	CD45.1 PerCP-Cy5.5 (clone A20)	BioLegend	110728	AB_893348
	CD45.2 V450 (clone 104)	eBioscience	48-0454-82	AB_11042125
	CD3 PE (clone 17A2)	BioLegend	100206	AB_312663
	CD11b FITC (clone M1/70)	BioLegend	101206	AB_312789
	B220 APC-Fire810 (clone RA3-6B2)	BioLegend	103277	AB_2860603
	Ter119 APC (clone TER-119)	eBioscience	17-5921-83	AB_469473
	Ghost Violet510 (clone N/A)	Tonbo Bioscience	50-105-2993	NA
	CD71 PE-Cy7 (clone RI7217)	BioLegend	113812	AB_2203382
7D-G, SF7A-C	CD45 BUV805 (clone 30-F11)	eBioscience	368-0451-82	AB_2896112
	IgD PB (clone 11-26c.2a)	BioLegend	405712	AB_1937244
	PD-1 BV421 (clone 29F.1A12)	BioLegend	135217	AB_2562568
	Ghost Violet510 (clone N/A)	Tonbo Bioscience	50-105-2993	NA
	CD11c BV570 (clone N418)	BioLegend	117331	AB_10900261
	CD45.2 BV605 (clone 104)	BioLegend	109841	AB_2563485
	CD45 BV650 (clone 30-F11)	BioLegend	103151	AB_2565884
	CXCR5 BV711 (clone L138D7)	BioLegend	145529	AB_2734207
	CD86 BV785 (clone GL-1)	BioLegend	105043	AB_2566722
	Ly6G FITC (clone 1A8)	BD Pharmingen	561106	AB_394207
	CD8a Spark Blue 550 (clone 53-6.7)	BioLegend	100780	AB_2819773
	CD11b PerCP-Cy5.5 (clone M1/70)	BioLegend	101228	AB_893232
	CD3 PE (clone 17A2)	BioLegend	100206	AB_312663
	CXCR3 PE-Dazzle594 (clone CXCR3-173)	BioLegend	126533	AB_2566563
	CD69 PE-Cy5 (clone H1.2F3)	BioLegend	104510	AB_313112
	CD44 PE-Cy7 (clone IM7)	BioLegend	103030	AB_830787
	Ly6C APC (clone HK1.4)	eBioscience	17-5932-82	AB_1724153
	CD4 Spark NIR 685 (clone GK1.5)	BioLegend	100476	AB_2819770
	MHCII AF700 (clone M5/114.15.2)	BioLegend	107622	AB_493727
	CD62L APC-Efluor780 (clone MEL-14)	eBioscience	47-0621-82	AB_1603256
	B220 APC-Fire810 (clone RA3-6B2)	BioLegend	103277	AB_2860603

Supplemental Table 4. Myeloid flow cytometry antibody panels

Myeloid Panels

Figures	Reagent	Source	Catalog #	RRID
7E-G	CD11b BUV805 (clone M1/70)	eBioscience	368-0112-82	AB_2896082
	F4/80 BV421 (clone BM8)	BioLegend	123131	AB_2563102
	Ghost Violet510 (clone N/A)	Tonbo Bioscience	50-105-2993	NA
	Ly6C BV570 (clone HK1.4)	BioLegend	128029	AB_10896061
	CD45.2 BV605 (clone 104)	BioLegend	109841	AB_2563485
	CD45 BV650 (clone 30-F11)	BioLegend	103151	AB_2565884
	CD86 BV785 (clone GL-1)	BioLegend	105043	AB_2566722
	Ly6G FITC (clone 1A8)	BD Pharmingen	561106	AB_394207
	CD11c SparkBlue 550 (clone N418)	BioLegend	117365	AB_2860633
	Siglec-H PerCP-Cy5.5 (clone 551)	BioLegend	129614	AB_10643995
	SIRPa PE (clone P84)	BioLegend	144012	AB_2563550
	CD45.1 PE-Cy7 (clone A20)	BioLegend	110730	AB_1134168
	Siglec-F APC (clone S17007L)	BioLegend	155508	AB_2750237
	CD19 SparkNIR 685 (clone 6D5)	BioLegend	115567	AB_2819828
	MHCII Alexa700 (clone M5/114.15.2)	BioLegend	107622	AB_493727
	XCR1 APC-Cy7 (clone ZET)	BioLegend	148224	AB_2783118
	B220 APC-Fire810 (clone RA3-6B2)	BioLegend	103277	AB_2860603

Supplemental Table 5. B cell flow cytometry antibody panels

B cell Panels

Figures	Reagent	Source	Catalog #	RRID
2A-B	CD138 BV421 (clone 281-2)	BioLegend	142507	AB_2565621
	MHCII Pacific Blue (clone M5/114.15.2)	BioLegend	107620	AB_493527
	Ghost Violet510 (clone N/A)	Tonbo Bioscience	50-105-2993	NA
	CD45.1 BV570 (clone A20)	BioLegend	110733	AB_10895765
	Fas BV605 (clone SA367H8)	BioLegend	152612	AB_2728202
	CD45 BV650 (clone 30-F11)	BioLegend	103151	AB_2565884
	CD21 BV711 (clone 4E3)	BioLegend	123435	AB_2876440
	CD86 BV785 (clone GL-1)	BioLegend	105043	AB_2566722
	GL7 FITC (clone GL7)	BioLegend	144603	AB_2561696
	CD11c SparkBlue550 (clone N418)	BioLegend	117365	AB_2860633
	CD45.2 PerCP-Cy5.5 (clone 104)	BioLegend	109828	AB_893350
	IgM PE (clone II/41)	eBioscience	12-5790-82	AB_465940
	CD69 PE-Cy5 (clone H1.2F3)	BioLegend	104510	AB_313112
	CD23 PE-Cy7 (clone B3B4)	eBioscience	25-0232-82	AB_469604
	AA4.1 APC (clone AA4.1)	BioLegend	136509	AB_2275879
	IgD AF700 (clone 11-26c.2a)	BioLegend	405712	AB_1937244
	CD19 APC-Cy7 (clone 6D5)	BioLegend	115529	AB_830706
	B220 APC-Fire810 (clone RA3-6B2)	BioLegend	103277	AB_2860603
3B-K, SF1-2, ST1	CD19 BUV737 (clone 6D5)	eBioscience	367-0193-82	AB_2895945
	CD138 BV421 (clone 281-2)	BioLegend	142507	AB_2565621
	MHCII Pacific Blue (clone M5/114.15.2)	BioLegend	107620	AB_493527
	Ghost Violet510 (clone N/A)	Tonbo Bioscience	50-105-2993	NA
	CD45.1 BV570 (clone A20)	BioLegend	110733	AB_10895765
	Fas BV605 (clone SA367H8)	BioLegend	152612	AB_2728202
	CD45 BV650 (clone 30-F11)	BioLegend	103151	AB_2565884
	CD21 BV711 (clone 4000)	BioLegend	123435	AB_2876440
	CD45.2 BV750 (clone 104)	BioLegend	109857	AB_2832376
	CD86 BV785 (clone GL-1)	BioLegend	105043	AB_2566722
	IgM FITC (clone II/41)	eBioscience	11-5790-81	AB_465244
	IgMa PerCP-Cy5.5 (clone MA-69)	BioLegend	408612	AB_2728436
	GL7 PE (clone GL7)	BioLegend	144608	AB_2562926
	CD69 PE-Cy5 (clone H1.2F3)	BioLegend	104510	AB_313112
	CD23 PE-Cy7 (clone B3B4)	eBioscience	25-0232-82	AB_469604
	AA4.1 APC (clone AA4.1)	BioLegend	136509	AB_2275879
	IgD AF700 (clone 11-26c.2a)	BioLegend	405712	AB_1937244
	CD11c APC-Cy7 (clone N418)	BioLegend	117324	AB_830649
	B220 APC-Fire810 (clone RA3-6B2)	BioLegend	103277	AB_2860603

Supplemental Table 6 T cell flow cytometry antibody panels

T cell panel

Figures	Reagent	Source	Catalog #	RRID
4B-K, SF3-4, ST2	TCRβ BUV737 (clone H57-597)	eBioscience	367-5961-82	AB_2896026
	CD127 BV421 (clone A7R34)	BioLegend	135023	AB_2563103
	Ghost Violet510 (clone N/A)	Tonbo Bioscience	50-105-2993	NA
	CD45.1 BV570 (clone A20)	BioLegend	110733	AB_10895765
	CD44 BV605 (clone IM7)	BioLegend	103047	AB_2562451
	CD45 BV650 (clone 30-F11)	BioLegend	103151	AB_2565884
	CXCR5 BV711 (clone L138D7)	BioLegend	145529	AB_2734207
	CD45.2 BV750 (clone 104)	BioLegend	109857	AB_2832376
	Tim3 BV785 (clone RMT3-23)	BioLegend	119725	AB_2716066
	TCR VA2 AF488 (clone B20.1)	BioLegend	127820	AB_2687230
	TCRgd PerCP-Cy5.5 (clone GL3)	BioLegend	118118	AB_10612756
	CD3 PE (clone 17A2)	BioLegend	100206	AB_312663
	CXCR3 PE-Dazzle594 (clone CXCR3-173)	BioLegend	126533	AB_2566563
	CD69 PE-Cy5 (clone H1.2F3)	BioLegend	104510	AB_313112
	CD8a PE-Cy7 (clone 53-6.7)	BioLegend	100722	AB_312761
	PD-1 PE-Fire810 (clone 29F.1A12)	BioLegend	135253	AB_2910293
	TCR VB5 APC (clone MR9-4)	BioLegend	139506	AB_10933250
	CD4 AF700 (clone RM4-5)	BioLegend	100536	AB_493701
	CD62L APC-efluor780 (clone MEL-14)	eBioscience	47-0621-82	AB_1603256

Supplemental Table 7. Primary immunofluorescence antibody panels

Figures	Reagent	Source	Catalog #	RRID
7C	Rabbit IgG anti-mouse CD3 (clone SP7)	Abcam	ab16669	AB_443425
	Goat IgG anti-mouse LYVE-1 (Polyclonal)	RND	AF2125	AB_2297188
	Rat IgG anti-mouse B220 Biotin (clone RA3-6B2)	BioLegend	103203	AB_312988
2C	Rat IgG anti-mouse B220 (clone RA3-6B2)	BD	550286	AB_393581
	PNA Biotin	Vector Labs	B-1075-5	NA

Supplemental Table 8. Secondary immunofluorescence antibodies

Figures	Reagent	Source	Catalog #	RRID
2C	Goat IgG anti-Rat IgG AF488 (polyclonal)	Invitrogen	A-11006	AB_2534074
	Goat IgG anti-Rabbit IgG AF555 plus (polyclonal)	Invitrogen	A32732	AB_2633281
	Streptavidin AF647	Invitrogen	S21374	NA
7C	Donkey IgG anti-Rabbit IgG AF488 (polyclonal)	Jackson	A48269	AB_2893137
	Donkey IgG anti-Goat IgG AF555 (polyclonal)	Invitrogen	A-21432	AB_2535853
	Streptavidin AF647	Invitrogen	S21374	NA
5A-B, 6A	Goat IgG anti-mouse IgG Dylight 549 (polyclonal)	Jackson	115-586-003	AB_2338890
	Goat IgG anti-mouse IgM Dylight 488 (polyclonal)	Invitrogen	SA5-10150	AB_2556730
