# Supplementary material for: Activation of autoreactive lymphocytes in the lung by radioresistant cells expressing a STING gain-of-function mutation
Source: JCI Insight. 2024 Jul 18;9(16):e174331. doi: 10.1172/jci.insight.174331 (PMC11343592; doi:10.1172/jci.insight.174331)
Supplement: Unedited blot and gel images [file jciinsight-9-174331-s009.pdf]

Unedited/Uncropped Gels

Full unedited gel for Figure 6B

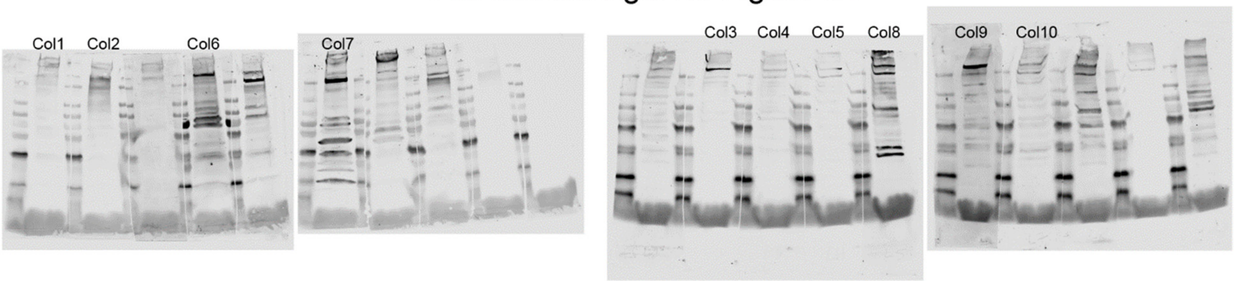

Full unedited gels for Figure 6C

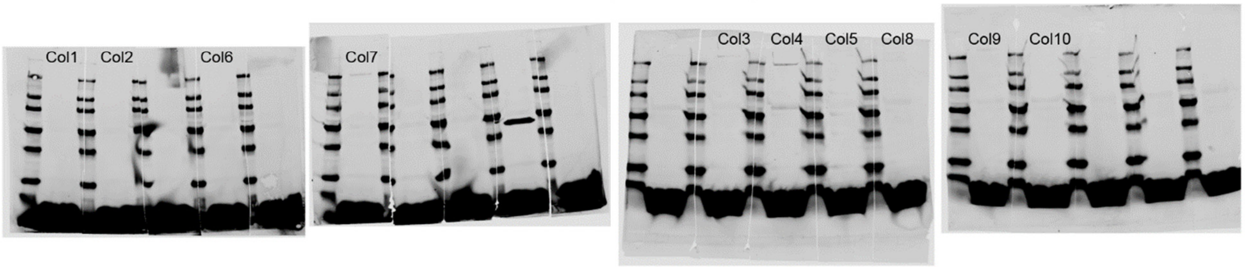

Lanes used for each figure are labelled with the column (Col) number they are featured as in the figure.

The unedited gels were developed as described in the methods section titled “Lung lysate western blots”. The gel was loaded and run with PageRuler Plus stained protein ladder and lung lysate, alternating. The resulting membrane was cut vertically at the lanes containing ladder so that each lane containing lung lysate could be stained with a primary antibody solution using sera from a unique biologic replicate of a chimeric mouse or a mouse IgG_1 anti-human β-actin monoclonal antibody control (diluted to 1:10,000, Biorad Cat #VMA0048).

For lanes run with lung lysate, the identity of the primary antibody solution going from left to right: WT→WT, WT→WT, WT→WT, WT→VM, WT→VM, WT→VM, WT→VM, WT→VM, anti-β-actin, no primary stain, WT→WT, WT→WT, WT→WT, WT→WT, WT→VM, WT→VM, WT→VM, WT→VM, WT→WT, WT→VM.
